# Genetic etiology of progressive pediatric neurological disorders

**DOI:** 10.1038/s41390-023-02767-z

**Published:** 2023-08-10

**Authors:** Juho Aaltio, Anna Etula, Simo Ojanen, Virginia Brilhante, Tuula Lönnqvist, Pirjo Isohanni, Anu Suomalainen

**Affiliations:** 1 Research Programs Unit, Stem Cells and Metabolism, University of Helsinki, Helsinki, Finland; 2 Department of Veterinary Biosciences, University of Helsinki, Helsinki, Finland; 3 Department of Child Neurology, Children’s Hospital, Pediatric Research Center, University of Helsinki and Helsinki University Hospital, Helsinki, Finland; 4 HUS Diagnostic Centre, Helsinki University Hospital, Helsinki, Finland; 5 HiLife, University of Helsinki, Helsinki, Finland

## Abstract

**Background:**

The aim of the study was to characterize molecular diagnoses in patients with childhood-onset progressive neurological disorders of suspected genetic etiology.

**Methods:**

We studied 48 probands (age range from newborn to 17 years old) with progressive neurological disorders of unknown etiology from the largest pediatric neurology clinic in Finland. Phenotypes included encephalopathy (54%), neuromuscular disorders (33%), movement disorders (11%), and one patient (2%) with hemiplegic migraine. All patients underwent whole-exome sequencing and disease-causing genes were analyzed.

**Results:**

We found 20 (42%) of the patients to have variants in genes previously associated with disease. Of these, 12 were previously reported disease-causing variants, whereas eight patients had a novel variant on a disease-causing gene: *ATP7A*, *CHD2*, *PURA*, *PYCR2*, *SLC1A4*, *SPAST*, *TRIT1*, and *UPF3B*. Genetics also enabled us to define atypical clinical presentations of Rett syndrome (*MECP2*) and Menkes disease (*ATP7A*). Except for one deletion, all findings were single-nucleotide variants (missense 72%, truncating 22%, splice-site 6%). Nearly half of the variants were de novo.

**Conclusions:**

The most common cause of childhood encephalopathies are de novo variants. Whole-exome sequencing, even singleton, proved to be an efficient tool to gain specific diagnoses and in finding de novo variants in a clinically heterogeneous group of childhood encephalopathies.

**Impact:**

- Whole-exome sequencing is useful in heterogeneous pediatric neurology cohorts.
- Our article provides further evidence for and novel variants in several genes.
- De novo variants are an important cause of childhood encephalopathies.

## Introduction

Pediatric neurological disorders are characterized with variable phenotypes, severity of symptoms, age at onset, and disease progression. Compared to adults, the developing nervous system and growth of the child sometimes mask classical neurological disease signs and symptoms are often unspecific, such as hypotonia.^1^ Furthermore, one gene can cause several different phenotypes, challenging the diagnostic process.^2^ Next-generation sequencing methods have greatly improved diagnostic accuracy in these heterogeneous disorders.^3^ Especially whole-exome sequencing (WES), which focuses on protein encoding regions of the genome, has been widely applied to pediatric patient cohorts^4^ and has become part of clinical diagnostics. Yet the success, often measured as diagnostic yield of WES, varies in different studies depending on the patient cohort phenotype and selection criteria. According to a recent meta-analysis, the average diagnostic yield of WES has been around 40% in all studies, with pediatric cohorts and studies with acute illness or neurological disorder as the test indication improving the yield.^3^ Achieving a molecular diagnosis has several benefits to the patient and their families: the possibility of a specific therapy, aid to family planning, and the conclusion to the diagnostic odyssey many patients must endure.

The aim of this study was to unravel the genetic etiologies of children with childhood-onset, unknown progressive neurological disorders in the largest pediatric neurology clinic in Finland. Our clinically mixed cohort of 48 children underwent WES. We found that WES, also for analyzing single probands and de novo variants, is a useful and effective diagnostic tool in this type of pediatric population, facilitating diagnosis of atypical presentations of classical syndromes.

## Materials and methods

### Ethics

The Ethics Review Board of the Hospital District of Helsinki and Uusimaa granted ethical approval for the study. Informed written consents were gathered from parents of child participants in accordance with the Declaration of Helsinki.

### Study population

The procedure for recruiting patients and the study population have been previously described in Aaltio et al.^5^ To summarize, patients with a severe neurological disorder of infancy or childhood-onset progressive neurological disorder were recruited ongoingly, with special attempt to enroll them already early in their diagnostic investigations, by experienced pediatric neurologists in the Children’s Hospital of Helsinki University Hospital, a tertiary care center unit, during 2015–2018. Patients with a known genetic disorder, clinically recognizable disorder, non-progressive intellectual disability, or autistic spectrum disorder were not considered. A total of 48 probands were recruited from 47 families, including one sister-pair. Of the families, 90% were of Finnish, 8% African, and 2% of other European origin. In one family, the parents were first-degree cousins. Of the probands 62% were male with a median age at inclusion of 5.7 years (range 0–17 years). Patient phenotypes included encephalopathy (54%), neuromuscular disorders (33%), and movement disorders (11%), while one patient had a varying phenotype: severe hemiplegic migraine. Beyond the study subjects, four families had more than one affected individual. Table 1 compiles the study population demographics.

**Table 1:** Study population demographics and distribution of genetic diagnoses.

Pedigree	Total	Confirmed diagnosis
Families	47 (100%)	19 (40%)
Consanguinity	1 (2%)	1 (100%)
One affected child	43	17 (40%)
Two or more affected children	4	3 (75%)
Sex		
Male	30 (62%)	12 (40%)
Female	18 (38%)	8 (44%)
Ethnicity		
Finnish	43 (90%)	17 (40%)
African	4 (8%)	3 (75%)
European non-Finnish	1 (2%)	0

### Whole-exome sequencing and variant analysis

Exome sequencing was performed to 48 probands following the methods as described in Sainio et al.^6^ The variant calling pipeline of the Finnish Institute of Molecular Medicine (FIMM) was used for the reference genome alignment and variant calling.^7^ Variants were analyzed primarily by suspected mode of inheritance, cross-referencing against variant databases^8,9^ and by bioinformatical prediction tools: Combined Annotation Dependent Depletion (CADD) C-score,^10^ PolyPhen,^11^ and SIFT.^12^ Variants with allele frequencies lower than 1% were prioritized. Pathogenicity of novel sequence variants was assessed according to ACMG criteria.^13^ Every patient had a phenotype highly specific for a disease of genetic etiology, and all reported novel sequence variants had computational evidence of deleteriousness and resided on highly conserved amino acid residues. We reported only disease-causing variants, not variants of uncertain significance or variants that could not be confirmed as disease-causing.

### Confirmation of findings

Most of the findings were confirmed by us with another independent method, Sanger sequencing, while all of them were confirmed by a clinical laboratory certified in genetic testing. In novel findings, Sanger sequencing was used to define variant segregation in the families. Paternity and maternity were confirmed by DNA fingerprinting to validate variant de novo status. For one family (P16 and P17), solid-phase minisequencing, a quantitative single-nucleotide detection assay,^14^ was used to confirm mosaicism in the family. Minisequencing is a method that permits quantifying the share of a single nucleotide variant in samples. The primers used for Sanger sequencing, minisequencing and parental testing, are reported in Supplementary Information.

## Results

### Genetic diagnoses in cohort

A total of 20 (42%) patients received a definitive molecular diagnosis by WES. Of the phenotypic groups, half of the patients with encephalopathy (*n* = 13) were diagnosed, while the proportions were smaller in the other groups: a third of patients with neuromuscular disorders (*n* = 5), and one out of five in the movement disorder group. Most findings indicated a dominant disorder (13/20) while autosomal recessive (AR) and X-linked recessive (XLR) disorders were only found in the encephalopathy group, five of the former and two the latter. Of the dominantly inherited disorders, the only sister-pair (P16 and P17) included in the study was diagnosed with a previously reported autosomal dominant (AD) missense variant in *DNM2*, causing Charcot–Marie–Tooth disease.^15^ The parents of the sisters were unaffected, initially eluding the inheritance pattern: Sanger sequencing was inconclusive, however pointing towards the mother being an unaffected carrier of the allele. Minisequencing performed on the mother’s blood, saliva, and urine revealed that the mother is a mosaic carrier, with a mutation load of 20–32% (see Supplementary Fig. 1 summarizing the findings of the family). A third patient had inherited a *GDAP1* variant from his asymptomatic mother; however, there were five other maternal family members that were affected, carried the variant, and received a molecular diagnosis for their disease. The fourth inherited AD variant was in *ATP1A2* causing childhood-onset hemiplegic migraine, affecting also the proband’s sister, father, and two other relatives. All the remaining inherited AD variants were de novo (7/11), including two unrelated patients with variants in the *PURA* gene (the patients have also been described in Reijnders et al.^16^). In addition, two X-linked dominant (XLD) variants were de novo, raising the total share of de novo mutations to 47%. One of the patients, P10, a girl of 2 years who had undergone extensive investigations for undetermined encephalopathy, with symptoms of vomiting, growth retardation, developmental delay, and lactic acidosis, had an XLD variant in *MECP2* causing Rett syndrome. Of the maternally inherited XLR variants, patient P04 with *UPF3B* variant had an affected brother with the same genetic finding, while the hemizygote *ATP7A* variant of patient P03 was absent in other male family members. Of the five AR disorders, two of the children were of African descent, the other with consanguineous parents, and one child manifested with a Finnish disorder, the PEHO syndrome^17^ caused by *ZNHIT3*. Table 2 compiles all patients with genetic diagnoses and the Mendelian Inheritance of Man (MIM) names of disorders.

**Table 2:** Molecular diagnoses discovered by WES.

Phenotype class	ID	Ethnicity	Sex/age at WES	Gene	IP	Variant	Zygosity	MIM diagnosis
Encephalopathy, *n* = 13/26	P01	FIN	M/8 m	*SLC1A4*	AR	c.1421 T > C, p.L474P^#^	Hom	#616657, Spastic tetraparesis, thin corpus callosum and progressive microcephaly
	P02	FIN	F/1 y	*TRIT1*	AR	c.70 C > T, p.P24S^#^ c.979 C > T, p.R327X^19,20^	Cmpd	#617873, COXPD35; encephalopathy, epilepsy, microcephaly
	P03	FIN	M/2 y	*ATP7A*	XLR	c.412 C > T, p.Q138X^#^	Hem	#309400, Menkes Disease
	P04	AFR	M/2 y	*UPF3B*	XLR	c.462_465delCGAT, p.I154MfsX20^#^	Hem	#300676, Mental retardation XLS14
	P05	FIN	M/3 y	*CHD2*	AD^DN^	c.1312 C > T, p.Q438X^#^	Het	#615369, Epileptic encephalopathy
	P06	AFR	F/3 y	*PYCR2*	AR	c.596 G > A, p.R199Q^#^	Hom	#616420, Leukodystrophy, hypomyelinating, 10
	P07	FIN	M/6 y	*PURA*	AD^DN^	c.299 T > G, p.L100R^#^	Het	#616158, Mental retardation AD31
	P08	FIN	F/2 m	*ZNHIT3*	AR	c.92 C > T, p.S31L^17^	Hom	#260565, PEHO syndrome
	P09	FIN	F/1 y	*PURA*	AD^DN^	c.289 A > G, p.K97E^25^	Het	#616158, Mental retardation AD31
	P10	FIN	F/2 y	*MECP2*	XLD^DN^	c.763 C > T, p.R255X^39^	Het	#312750, Rett syndrome
	P11	FIN	M/4 y	*ATP1A3*	AD^DN^	c.2324 C > T, p.P775L^40^	Het	#601338, CAPOS syndrome
	P12	AFR	F/4 y	*ERCC8*	AR	c.551–1 G > A^41^	Hom	#216400, Cockayne syndrome type A
	P13	FIN	M/6 y	*PDHA1*	XLD^DN^	c.757 A > G, p.R253G^42^	Hem	#312170, Pyruvate dehydrogenase deficiency
Neuromuscular disorders, *n* = 5/16	P14	FIN	M/1 y	*SPAST*	AD^DN^	c.1166 C > T, p.T389I^#^	Het	#182601, Spastic paraplegia 4
	P15	FIN	M/12 y	*GDAP1*	AD	c.368 A > G, p.H123R^43^	Het	#607831, Charcot–Marie–Tooth axonal type 2K
	P16	FIN	F/12 y	*DNM2*	AD	c.1072 G > A, p.G358R^15^	Het	#606482, Charcot–Marie–Tooth axonal type 2M
	P17	FIN	F/13 y	*DNM2*	AD	c.1072 G > A, p.G358R^15^	Het	#606482, Charcot–Marie–Tooth axonal type 2M
	P18	FIN	M/15 y	*SLC2A1*	AD^DN^	c.823 G > A, p.A275T^44^	Het	#612126, GLUT1 deficiency syndrome 2
Movement, *n* = 1/5	P19	FIN	M/14 y	*ADCY5*	AD^DN^	c.2176 G > A, p.A726T^45^	Het	#606703, Dyskinesia, familial, facial myokymia
Other, *n* = 1	P20	FIN	M/13 y	*ATP1A2*	AD	c.1816G>A, p.A606T^46^	Het	#104290, Alternating hemiplegia of childhood

*IP* inheritance pattern, *FIN* Finnish heritage, *AFR* African heritage, *AD* autosomal dominant, *AR* autosomal recessive, *XLR* X-linked Recessive, *XLD* X-linked dominant, *DN* de novo mutation, *#* novel finding, *Het* heterozygote, *Hom* homozygote, *Hem* hemizygote, *Cmpd* compound heterozygote.

### Novel findings in known disease-causing genes

#### Novel variant and possible Finnish founder allele in TRIT1

**Fig. 1:**
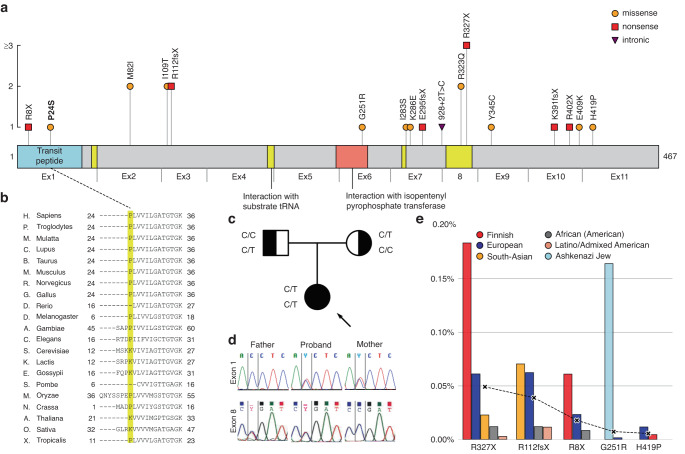
Summary of findings in TRIT1. **a** TRIT1 protein with domains according to Uniprot, and previously reported disease-causing variants, with exons indicated below the linear graph. The vertical axis depicts the number of patients found in literature, including the variants found in this study. Our novel variant p.P24S resides on the mitochondrial transit peptide, where one previous variant has been described. References for the gene review can be found in supplementary information. **b** Protein conservation through species of amino acid residue Pro24 (yellow shade) and bordering regions. **c** Family pedigree, both parents were unaffected carriers of different variants, the affected proband’s genotype compound heterozygote. **d** Sanger sequencing results of family, the first row depicting the maternally inherited c.70 C > T variant, the second row the paternally inherited c.979 C > T. **e** Of the previously reported 16 pathogenic variants, 11 are found in gnomAD as rare variants. The bar graph depicts the allele frequency of those five variants with total allele frequency greater than 0.005%, in different genetic subpopulations (bars), in order of total frequency (dotted line).

The proband, P02, was born at term uneventfully, with normal growth and no family history of hereditary disorders. At 4 months of age, she presented initially with vertical nystagmus, which later receded. At 9 months age, she suffered of seizures and was diagnosed with focal epilepsy, initially difficult to treat but later well in control with antiepileptic medication. Brain MRI at 4 and 9 months of age showed thin corpus callosum and white matter abnormality. She also had microcephaly, with her head circumference being −3 SD at 1 year of age, and significant spasticity in legs. She learned to crawl but lost that skill. At 7 years of age, she does not move independently or talk, but uses communication aids. WES revealed two variants on *TRIT1* (NM_017646.6), c.70 C > T (p.Pro24Ser) and c.979 C > T (p.Arg327*). The gene, tRNA isopentenyltransferase 1, is associated with combined oxidative phosphorylation deficiency 35 (COXPD35, MIM#617873).^18^ We collected previously reported disease-causing variants of *TRIT1* (Fig. 1a), and found that p.R327X has been previously reported as pathogenic by two independent studies,^19,20^ while p.P24S is novel, located on the mitochondrial transit peptide sequence, and conserved in species down to arthropods (Fig. 1b). The findings were confirmed by Sanger sequencing to have been inherited as compound heterozygous from the mother and father, respectively (Fig. 1c, d). The allele frequencies of disease associated *TRIT1* variants (Fig. 1e) showed that p.R327X is the most common variant, most common in Finns with an allele frequency of 0.18% (globally 0.05%). The p.P24S variant is not found in genomic databases. Histological analysis of skeletal muscle sample showed normal morphology, but reduced activity of respiratory chain complex IV and low amount of complex I, fitting to a mitochondrial translation defect. The novel p.P24S replaces a hydrophobic proline with a polar serine in the mitochondrial targeting sequence, suggesting reduced entry of the mutant protein to mitochondria. Since the patient’s phenotype corresponds to previous descriptions of *TRIT1* caused COXPD35 disease, the variant p.P24S was found in trans with a known pathogenic variant, and it affects a conserved amino acid altering the protein’s hydrophobicity, p.P24S was judged to be likely pathogenic (ACMG criteria and computational evidence of pathogenicity in Table 3).

**Table 3:** Novel variants in previously known disease-causing genes, phenotypes by HPO terms, ACMG guidelines variant classification.

Gene	Chromosomal position (GRCh37/hg19)	Variant	CADD *C*-score	PolyPhen prediction	SIFT prediction	gnomAD AF Total	gnomAD AF Finns	Inherit.	Phenotype by HPO terms	ACMG guidelines
*ATP7A*	chrX:g.77244029 C > T	c.412 C > T p.Q138X	36.0	–	–	–	–	XLR	Encephalopathy, central hypotonia, athetosis	Pathogenic PVS1; PM2; PP3,4
*CHD2*	chr15:g.93489381 C > T	c.1312 C > T p.Q438X	38.0	–	–	–	–	De novo	Generalized myoclonic seizures, abnormality of the cerebellar vermis, global developmental delay	Pathogenic PVS1; PM2,6; PP3,4
*PURA*	chr5:g.139494065 T > G	c.299 T > G p.L100R	27.0	Probably damaging 0.978	Deleterious 0.00	–	–	De novo	Intellectual disability, epilepsy, ventricular septal defect, retinoblastoma, sleep apnea	Likely pathogenic PM2,5,6; PP3,4
*PYCR2*	chr1:g.226109289 C > T	c.596 G > A p.R199Q	32.0	Possibly damaging 0.812	Deleterious low confidence 0.00	0.0100%	–	AR	Global developmental delay, dystonia, fasciculations, delayed myelination, ataxia, hypoplasia of corpus callosum	Likely pathogenic PM2,5; PP3,4
*SLC1A4*	chr2:g.65248102 T > C	c.1421 T > C p.L474P	29.4	Probably damaging 1.000	Deleterious 0.00	0.0018%	0.0040%	AR	CNS hypomyelination, microcephaly, epilepsy, global developmental delay, hypoplasia of corpus callosum, spastic tetraplegia	Likely pathogenic PM1,2; PP3,4
*SPAST*	chr2:g.32352084 C > T	c.1166 C > T p.T389I	27.4	Probably damaging 0.999	Deleterious 0.04	–	–	De novo	Spastic paraparesis	Pathogenic PS2; PM1,2,5; PP3,4
*TRIT1*	chr1:g.40349094 G > A	c.70 C > T p.P24S	24.6	Possibly damaging 0.539	Deleterious 0.02	–	–	AR	Nystagmus, progressive microcephaly, leukoencephalopathy, epilepsy, global developmental delay,	Likely pathogenic PM2,3; PP3,4
*UPF3B*	chrX:g.118979164CGAT/-	c.462_465delCGAT p.I154MfsX20	34.0	–	–	–	–	XLR	Infantile muscular hypotonia, global developmental delay, autistic behavior	Pathogenic PVS1; PM2; PP1,3,4

*AF* allele frequency, *CADD* Combined Annotation Dependent Depletion, *gnomAD* Genome Aggregation Database, *SISu* Sequencing Initiative Suomi, *SIFT* Sorting Intolerant From Tolerant tool, *ACMG* American College of Medical Genetics and Genomics, *PVS* pathogenic very strong, *PS* pathogenic strong, *PM* pathogenic moderate, *PP* pathogenic supporting.

#### SLC1A4 caused spastic tetraparesis, thin corpus callosum, and progressive microcephaly

**Fig. 2:**
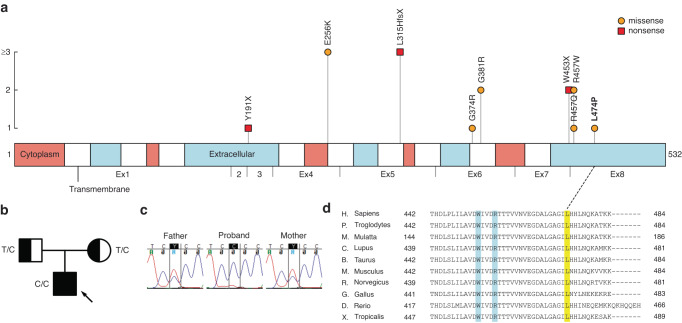
Summary of findings in SLC1A4. **a** SLC1A4 protein with domains according to Uniprot, and previously reported disease-causing variants, with exons indicated below the linear graph. The vertical axis depicts the number of patients found in literature, including the variant found in this study. Our novel variant p.L474P resides on the 8th exon, where two previous variants have been described. References for the gene review can be found in supplementary information. **b** Family pedigree: the unaffected parents were both heterozygote carriers and the affected proband homozygote. **c** Sanger sequencing results, both parents were heterozygote for the c.1421 T > C variant, the proband homozygote. **d** Protein conservation through species of amino acid residue Leu474 (yellow shade) and previously reported variants (blue shade).

The proband, P01, was the first child of the family, born with microcephaly −2.45 SD, but otherwise healthy. At 7 months of age, he began having tonic seizures. EEG showed multifocal spikes, increasing during sleep. Brain MRI at 8 months showed hypomyelination, a thin corpus callosum, gray matter abnormality, and mildly enlarged ventricles. He had spastic tetraparesis, more prominently in the upper limbs and shoulder region. At the age of 1 year and 4 months, his microcephaly was remarkable, with his head circumference being −7.7 SD. He had cerebral visual impairment, did not appear to take any contact during clinical examination and could not move independently. WES revealed a novel homozygote *SLC1A4* (NM_003038.5) missense variant c.1421 T > C (p.Leu474Pro). SLC1A4 is an L-serine transporter associated with spastic tetraparesis, thin corpus callosum, and progressive microcephaly (SPATCCM, MIM#616657). We collected previously reported disease-causing variants of *SLC1A4* (Fig. 2a), and found that the variant resides on exon 8, associated with two previously reported variants. Sanger sequencing confirmed both the parents to be heterozygote carriers (Fig. 2b, c). The variant resides on a highly conserved amino acid position (Fig. 2d), it is very rare and does not appear as homozygote in genomic databases (allele frequency highest in Finns 0.0040%, total 0.0018%). The variant was classified as likely pathogenic (ACMG criteria and further computational evidence of pathogenicity in Table 3). Empirical experiences suggested usefulness of L-serine supplementation in SPATCCM,^21^ and the patient was begun L-serine substitution as a treatment trial. During the treatment, no improvement was seen clinically in the proband’s symptoms nor in an EEG control, and the medication was discontinued after 2 months.

#### An atypical, less-severe Menkes disease

**Fig. 3:**
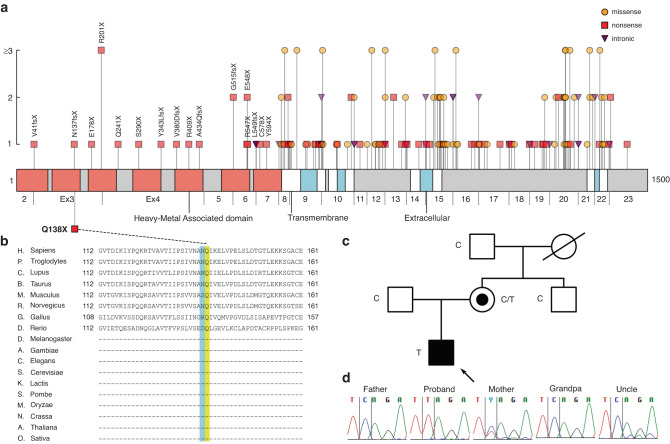
Summary of findings in ATP7A. **a** ATP7A protein with domains according to Uniprot, and previously reported disease-causing variants, with exons indicated below the linear graph. The vertical axis depicts the number of patients found in literature. Protein level consequences shown for truncating variants in exons 2–7. Our novel variant p.Q138X resides on the 3rd exon, on the second Heavy-Metal Associated domain. References for the gene review can be found in supplementary information. **b** Protein conservation through species of amino acid residue p.Gln138 (yellow shade) and previously reported variants (blue shade). **c** Family pedigree: the affected proband was hemizygote for the variant carried by the mother, while all other maternal relatives alive were not carries. **d** Sanger sequencing results of the proband, parents, maternal uncle, and grandfather. The mother is a heterozygote carrier of the X-chromosomal c.412 C > T variant, while the affected proband’s genotype is hemizygote.

The proband, P03, was the second child of the family, born uneventfully at term, with normal initial growth. There was no family history of hereditary conditions. At 8 months of age he stopped reaching new motoric milestones and presented with severe hypotonia. At 2 years of age, brain MRI showed cerebellar and vermis atrophy, with signal intensities in the thalamus and dentate nuclei. He had severe ataxia and athetosis. At 6 years of age, he was diagnosed with epilepsy, has not reached any new milestones, does not talk nor move independently, and is extremely hypotonic with athetoid movement. At 8 years of age, he required a gastrostomy. WES revealed a novel hemizygote *ATP7A* (NM_000052.7) truncating variant c.412 C > T (p.Gln138*). Mutations on *ATP7A*, the ATPase copper transporting alpha protein, cause Menkes disease (MIM#309400), a copper metabolism disorder leading to infant-onset encephalopathy, known by the coarse, kinky or steely hair on patients.^22^ We collected previously reported disease-causing variants (Fig. 3a), and found that nonsense variants are a common cause of the disease. The variant resided on a highly conserved amino acid residue (Fig. 3b) and is absent from human genome databases.

The proband’s phenotype did not match the classical Menkes disease presentation. Due to the genetic finding, the proband’s copper metabolism was assessed by measuring serum copper and ceruloplasmin (S-Cu 11 µmol/l, ref. 12.6–23.6 µmol/l; S-Ceruloplasmin 143 mg/l, ref. 200–500 mg/l), which were slightly below normal, supporting a copper metabolism disorder. A brain MRI controlled at 3 years of age showed previous findings, but also elongation and tortuosity of intracranial arteria, common in Menkes disease.^22^ Family samples available were tested for the variant by Sanger sequencing: the mother was a carrier of the X-chromosomal variant, whereas neither the proband’s unaffected maternal uncle nor grandfather had the variant (Fig. 3c, d). The variant was classified as pathogenic (ACMG criteria and computational evidence of pathogenicity in Table 3).

#### Childhood-onset severe hereditary spastic paraplegia

**Fig. 4:**
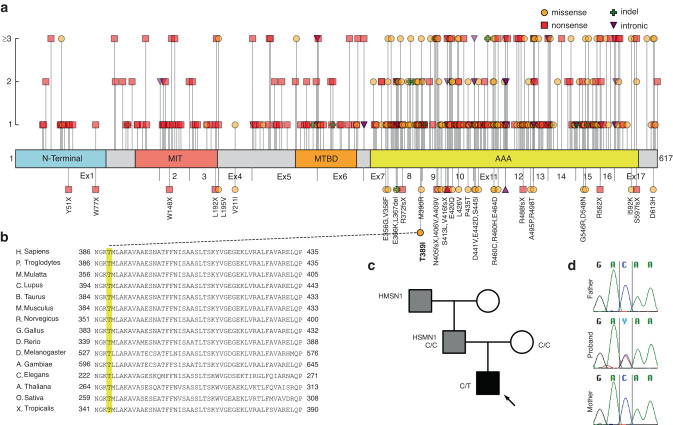
Summary of findings in SPAST. **a** SPAST protein with domains according to Uniprot, and previously reported disease-causing variants; the variants below the linear graph have been described in children. The vertical axis above depicts the number of patients found in literature. Our novel p.T389I variant resides on the AAA-cassette, considered a mutational hotspot. References for the gene review can be found in supplementary information. **b** The Thr389 amino acid residue, indicated with yellow shade, is highly conserved throughout species. **c** Family pedigree, the affected proband was found to have a de novo variant c.1166C>T. The father and his father were affected by a clinically diagnosed HSMN1. **d** Sanger sequencing confirmed the variant to be de novo. MIT microtubule interacting and trafficking, MTBD microtubule binding domain, AAA ATPase associated with diverse cellular activities.

The proband, P14, was the second child of the family, born uneventfully at term, with normal initial growth. His father had been clinically diagnosed with mild hereditary motor sensory neuropathy (HMSN1) that also affected his father and grandfather, but genetic testing, including WES, had been inconclusive. At 8 months of age, the proband could not sit up, and he learned to crawl first at 11 months of age. At clinical examination, the proband presented with severe lower limb spasticity. Brain and spine MRI, as well as electroneuromyography (ENMG), were all normal. The child has never been ambulatory. WES revealed a novel heterozygote *SPAST* (NM_014946.4) missense variant c.1166 C > T (p.Thr389Ile). We collected previously reported disease-causing variants (Fig. 4a), finding that the variant resides on the “ATPases Associated with diverse cellular Activities” (AAA) domain, showing clustering of SPAST-related pathogenic variants. Most variants cause adult-onset HSP, but there are some reports of childhood-onset disease. The variant p.T389I is not found in genomic databases and is highly conserved through species (Fig. 4b). Sanger sequencing as well as parental testing confirms it to be de novo (Fig. 4c, d). Another missense variant on the same amino acid site, p.Thr389Ala, has been reported in Clinvar (ID 188190) by multiple submitters as causing hereditary spastic paraplegia (HSP). The variant was classified as pathogenic (ACMG criteria and computational evidence of pathogenicity in Table 3), and the HSP-disease of the proband to be unrelated to HMSN1 in the family.

### Novel variants with typical phenotypes on previously disease-associated genes

We found novel variants on four more patients with typical geno-phenotypes (Table 3). Defects in Chromodomain Helicase DNA-Binding Protein 2 (*CHD2*) are known to cause developmental and epileptic encephalopathy 94 (MIM#615369), whereas Regulator of Nonsense-Mediated mRNA Decay (*UPF3B*) is associated with X-linked syndromic intellectual developmental disorder 14 (MIM#300676). For probands P05 and P04, both *CHD2* (NM_001271.4) c.1312 C > T (p.Gln438Ter) and *UPF3B* (NM_080632.3) c.462_465delCGAT (p.Ile154Metfs*20) are loss of function (LOF) variants in genes where LOF is a known mechanism of disease,^23,24^ and are as such classified as pathogenic (ACMG criteria in Table 3, additional data on Supplementary Figs. 2 and 3). Purine-Rich Element-Binding Protein A (*PURA*) causes a neurodevelopmental disorder with neonatal respiratory insufficiency, hypotonia, and feeding difficulties (MIM#616158), and Pyrroline-5-Carboxylate Reductase 2 (*PYCR2*) causes hypomyelinating leukodystrophy 10 (MIM#616420). For probands P07 and P06, both *PURA* (NM_005859.5) c.299 T > G (p.Leu100Arg) and *PYCR2* (NM_013328.4) c.596 G > A (p.Arg199Gln) resided on highly-conserved amino acid residues previously associated with other pathogenic missense variants, *PURA* (p.Leu100Pro)^25^ and *PYCR2* (p.Arg199Trp),^26^ and the variants were assigned as likely pathogenic (ACMG criteria in Table 3, additional data on Supplementary Figs. 4 and 5).

## Discussion

Here, we report genetic findings of progressive neurological disorders of children in a mixed cohort from the largest pediatric neurology clinic in Finland. In our patient cohort of 47 families, not including apparent known syndromes, 19 received a definitive genetic diagnosis. Most of the variants were sporadic and de novo born, further emphasizing the importance of next-generation sequencing techniques in the diagnostic process. We aimed also to further describe the genetic landscape of pediatric encephalopathies in Finland. While 90% of our patients were of Finnish heritage, all families carried their specific gene defects and only one patient had a Finnish disease heritage disorder, PEHO syndrome. This indicates that disorders of Finnish disease heritage are well identifiable and therefore not included in the cohort of diseases with unknown background.

Eight of the found variants were novel and have not been previously reported to the best of our knowledge. These include variants in *TRIT1*, *SPAST*, *ATP7A*, and *SLC1A4*. Only 17 patients have been reported with defects in *TRIT1* gene (MIM#617873), which encodes a tRNA isopentenyltransferase modifying mitochondrial tRNAs.^18–20^ In addition to the novel p.P24S variant, we present the first Finnish patient with the p.R327X^19,20^ variant, previously described in two studies from France^20^ and USA/China.^19^ The p.R327X is the most common pathogenic *TRIT1* variant in genomic databases, and three times more frequent in the Finnish population than in others, suggesting it might be a Finnish founder allele.

Only 20 published patients having spastic tetraparesis, thin corpus callosum and progressive microcephaly (SPATCCM) have been published with *SLC1A4* variants. This protein transports L-serine and participates in brain serine metabolism, importing L-serine to neuronal cells. SPATCCM is mostly reported in Ashkenazi Jews,^21^ with only three occurrences in European populations.^27–29^ Here we report the fourth European case of *SLC1A4* caused SPATCCM with the hallmark symptoms. The variant is very rare, and although its reported frequency is highest in Finns, the sample (n = 1) in gnomAD is insufficient for further conclusions. Based on empirical experience of potentially beneficial effects of L-serine supplementation in Ashkenazi patients, our patient underwent a treatment trial, but with no detectable effect.

*ATP7A* is an ATPase copper transporting alpha protein which causes Menkes syndrome. Our patient’s symptoms started late, at 8-months, and he is still alive at 8-years old. Typically, Menkes manifests at 2–3 months with life expectancy less than 4 years. Our patient’s variant p.Q138X lies in the third exon. Truncating variants in exons 3 and 4 have also previously been suggested to cause moderate phenotypes of Menkes disease due to translation reinitiation in exon 5, leading to a partially functional protein.^30^ Previous reports included frameshift variants p.Val41fsX and p.Asn137Lysfs*22; the latter manifesting at the same time as our patient, and both having less-severe phenotypes than typically.^30,31^ Our report supports the conclusion that truncating variants in exons 3 or 4 of *ATP7A* associate with a slightly delayed manifestation and progression of Menkes disease.

Mutations in *SPAST*, a member of the AAA protein family, are the most frequent cause of both sporadic and familial HSP (MIM#182601), but childhood-onset, such as that of our patient, is rare.^32^ Schieving et al.^33^ concluded recently that all pediatric *SPAST*-related phenotypes have been caused by de novo variants on the AAA-cassette of SPAST. This conclusion is supported by our finding. The pathogenicity of our patient’s variant is supported by its conservation and also the fact that a previous patient with childhood-onset HSP, manifesting at 1 year of age, had a de novo *SPAST* variant changing an amino acid residue adjacent to that of our patient (p.Met390Val^34^ vs our patient’s p.Thr389Ile). It has been proposed that early childhood-onset HSP caused by *SPAST* could be accompanied by other modifier variants in *SPAST* such as the p.Ser44Leu, and one of such variants is typically found on one of the parents.^33^ Our patient, however, does not have other variants, and the variant is de novo. With the age-at-onset of 8 months, our patient is one of the earliest-onset patients with the disease so far reported.

Our approach used singleton WES. Previously we have ascertained that such analysis is a cost-effective and efficient tool in the diagnostic workup.^5^ Here, families with more than one affected child were more likely to gain a diagnosis (75% success rate) than families with a single affected child (40% success; de novo mutation in 53% of the cases). Several studies have investigated the difference in performance between singletons and trios. A recent study with 700 patients investigated exomes with a mix of singletons, duos and trios, and found no considerable difference in the diagnostic yield.^4^ Another study examined the performance between singletons and trios in a double-blinded randomized exome cohort of comparable size to this study. Their trios provided one additional diagnosis over singletons.^35^ Trios are better at distinguishing de novo and biallelic variants in probands, but with higher costs. Our study suggests, however, that disease causing variants in children can usually be found with singleton WES with the help of in silico predictions, absence of the variant in genomic databases, and segregation analyses with Sanger sequencing. This applies particularly when the finding is a previously reported pathogenic variant with matching phenotype (60% of the diagnoses in our study). However, Sanger sequencing is required to investigate segregation patterns for completely novel variants. We analyzed seven additional candidate variants (for five patients) by Sanger sequencing, showing that these were neutral variants, inherited from parents or present in unaffected siblings. An eighth variant, in a phenotypically well-matched gene, previously reported pathogenic but with a dissimilar phenotype, could not be confirmed de novo due to a missing parental sample and was not reported.

WES reached a genetic diagnosis in 42% of the cases. The number is comparable to other studies using WES.^3^ The majority still remained undiagnosed, and the limitations of WES have been widely described in literature.^36,37^ For instance, WES does not cover intronic areas, and cannot reliably identify e.g. chromosomal rearrangements. In our study, most of the patients had chromosomal microarray done without actionable findings before study inclusion. CNVs were not analyzed in this study. Some disorders can be of polygenic origin, there could still be unrecognized disease-causing genes overlooked in the analysis, and sometimes, disorders are of other than genetic origin. In the case of children, progressive neurological disorders are however often of genetic origin compared to some adulthood onset disorders where lifestyle choices can affect disease presentation. Noteworthy, some of our patients had had years of extensive diagnostic workups including karyotyping, different gene tests and gene panels without success, and received their diagnosis only after WES. An interesting avenue of research is finding the best strategy in implementing WES to benefit most patients and decrease the number of undiagnosed. A recent consensus statement reported that WES should precede chromosomal microarrays in some neurological disorders in children due to higher diagnostic yields.^38^ Considering cost-effectiveness, Tan et al.^35^ find that due to the high chance of a positive singleton WES, it would be cost-effective to always start with singleton and only later escalate to a trio if needed. Wortmann et al.^37^ describe widely the pitfalls of WES and suggest a genomic checklist to help focusing and combining different lines of investigations after a negative WES. Finding the optimal strategy requires still research efforts, but as prices of sequencing are expected to decrease, there will be increased availability for WES.

To conclude, we provide novel variants and further evidence for several rare pathogenic genes. In line with previous exome cohorts with childhood-onset neurological disorders, de novo variants are an important cause of these disorders.^4^ Also, due to the clinical variability of pediatric disorders, even classical syndromes can be challenging to diagnose in early stages or when presenting atypically. In these cases WES provides an opportunity for early diagnosis by interrogating all genes at once. Singleton WES led to diagnosis of several family members in three families. WES also provided actionable, treatment-modifying effect for at least three patients (15% of the diagnosed, 6% of all): ketogenic diet was recommended for the patients with GLUT1 deficiency syndrome (MIM#612126; *SLC2A1*) and pyruvate dehydrogenase deficiency (#312170; *PDHA1*), and L-serine supplementation was initiated for the patient suffering of SPATCCM (MIM#616657*; SLC4A1*). According to our study, singleton WES is useful in diagnosing sporadic disorders.

### Supplementary information

Supplementary information

## Data Availability

The data that support the findings of this study are available upon reasonable request.
