# Supplementary material for: Genetic etiology of progressive pediatric neurological disorders
Source: Pediatr Res. 2023 Aug 10;95(1):102–11. doi: 10.1038/s41390-023-02767-z (PMC10798881; doi:10.1038/s41390-023-02767-z)
Supplement: Supplementary file 1 — Supplementary information [file 41390_2023_2767_MOESM1_ESM.pdf]

Supplementary file 1

Summary

This file contains supplementary material for the methods section, additional figures summarizing the findings of P16 and P17 (*DNM2*) and remaining patients with novel variants, and extensive references for the gene literature reviews.

Contents

1. List of primers ... 2
   - 1.1 Primers used in Sanger sequencing ... 2
   - 1.2 Primers for minisequencing DNM2 exon 8 ... 2
   - 1.3 Primers for paternity fingerprinting ... 3
2. Reference genome and gene transcripts ... 3
3. Graphics software ... 4
4. Supplementary figure 1: DNM2 ... 4
5. Supplementary figure 2: CHD2 ... 5
6. Supplementary figure 3: UPF3B ... 5
7. Supplementary figure 4: PURA ... 6
8. Supplementary figure 5: PYCR2 ... 6
9. Gene review references ... 7
   - 9.1 TRIT1 ... 7
   - 9.2 SLC1A4 ... 7
   - 9.3 ATP7A ... 7
   - 9.4 SPAST ... 8
   - 9.5 CHD2 ... 9
   - 9.6 UPF3B ... 9
   - 9.7 PURA ... 10
   - 9.8 PYCR2 ... 10
   - 9.9 DNM2 ... 10

1. List of primers

1.1 Primers used in Sanger sequencing

Chr	pos	Gene	Forward	Reverse
1	40349094	TRIT1_ex1	CTCCTCCCTAAGCAACCTCG	GGCTCCCTTTACCTACCCC
1	40312919	TRIT1_ex8	CCTCTCTGCAGGGTAAGGAG	AAAGATGCTGCTGTTCTTCTTAG
1	43395308	SLC2A1_ex6	CTTCTTCCCACTCTGAGCCA	CATGCACACTTGACCAGAGG
1	153296516	MECP2_ex3	AACCACCTAAGAAGCCCAAA	CTGCACAGATCGGATAGAAGAC
1	160100376	ATP1A2_ex13	CACTCTGCGGATCTCACTGA	ACTGCAGCTCCTTGAACTCT
1	226109289	PYCR2_ex5	GGGCTTAGTGAGTGTCTCCA	CTTCCCATACCCACTGCTCC
2	65248102	SLC1A4_ex8	TGACTTTCCTTCGGCATCCA	CAGGGGCGATGTCTCCTC
2	32352084	SPAST_ex8	CTATGGGCAGCTCTGTTTGG	GAGCCCAGATCACTCAAAACA
3	123038601	ADCY5_ex10	GTCAGCCTGTCTCACGCC	TTTTCTTGCCCTCCTCCTCC
5	60198336	ERCC8_ex7	TTGGCCTCACTTTCTTCAGAA	AGTTCCTCTGTGTTCTAAGGTGA
5	139494055	PURA_ex1	GAACAAGCGCTTCTACCTGG	TTCTCGCGCACCAGGAAC
8	75272429	GDAP1_ex3	TCTGGTGCATCAGGCCATTT	TCCGACTGGTTCATGGATCG
15	93489381	CHD2_ex12	AAGCCGGCACCCTCAAAT	GCGACACAGCGAAACTCT
17	34842784	ZNHIT3_ex1	GGAGAAGCCCAAATACCGCT	CGTCCAGCCTCCGTCTTG
19	10904475	DNM2_ex8	AACCCTGGCTTGACTTGGAA	CCACCACAAGTTCAGGCCTA
19	42474634	ATP1A3_ex17	GCAACACAGCGAGACTCT	TCCCCTGAGTCAATGCCAG
X	19373620	PDHA1_ex5	GGCAGCTTTGTGGAAATTACC	ACATGACCTGCTCCTGTATGA
X	77244029	APT7A_ex3	CACTGACTTTGCCATGGGAC	CACGACTTCACCAGCTTGAG
X	118979164	UPF3B_ex4	GGAGACCCTCTTCAGATGACT	GGGGTCGAATTAACTCTCACA

1.2 Primers for minisequencing DNM2 exon 8

Primer	Sequence
Biotinylated forward primer	GATCGAGGGCTCAGGAGATC
Reverse primer	CTTCACCAGCTCAAATGGGAA
Probe primer	GATTCGGGCGCCCC

1.3 Primers for paternity fingerprinting

D8S1179, D21S11, D16S539, D2S1338, D18S51, VWA, FGA

2. Reference genome and gene transcripts

**Reference genome:**	GRCh37
**Gene**	**RefSeq/Ensemble transcript**
ADCY5	ENST00000462833.1
ATP1A2	NM_000702.4
ATP1A3	NM_152296.5
ATP7A	NM_000052.7
CHD2	NM_001271.4
DNM2	NM_001005361.3
ERCC8	NM_000082.4
GDAP1	NM_018972.4
MECP2	NM_001110792.2
PDHA1	ENST00000422285.2
PURA	NM_005859.5
PYCR2	NM_013328.4
SLC1A4	NM_003038.5
SLC2A1	NM_006516.4
SPAST	NM_014946.4
TRIT1	NM_017646.6
UPF3B	NM_080632.3
ZNHIT3	NM_004773.4

3. *Graphics software*

All figures were rendered in Python[1] (version 3.8.5) with relevant modules[2-3] using Matplotlib[4].

References:

1. Van Rossum, G. & Drake, FL. *Python 3 Reference Manual*. 2009, Scotts Valley, CA: CreateSpace
2. McKinney, W. Data Structures for Statistical Computing in Python. *Proceedings of the 9th Python in Science Conference (SCIPY 2010)*, 2010:56-61
3. Harris CR, Millman KJ, van der Walt SJ, et al. Array programming with Numpy, *Nature*, 2010;585(7825):357-362
4. Hunter JD. Matplotlib, A 2D graphics environment. *Computing in Science & Engineering*, 2007;9(3):90-95

4. *Supplementary figure 1: DNM2*

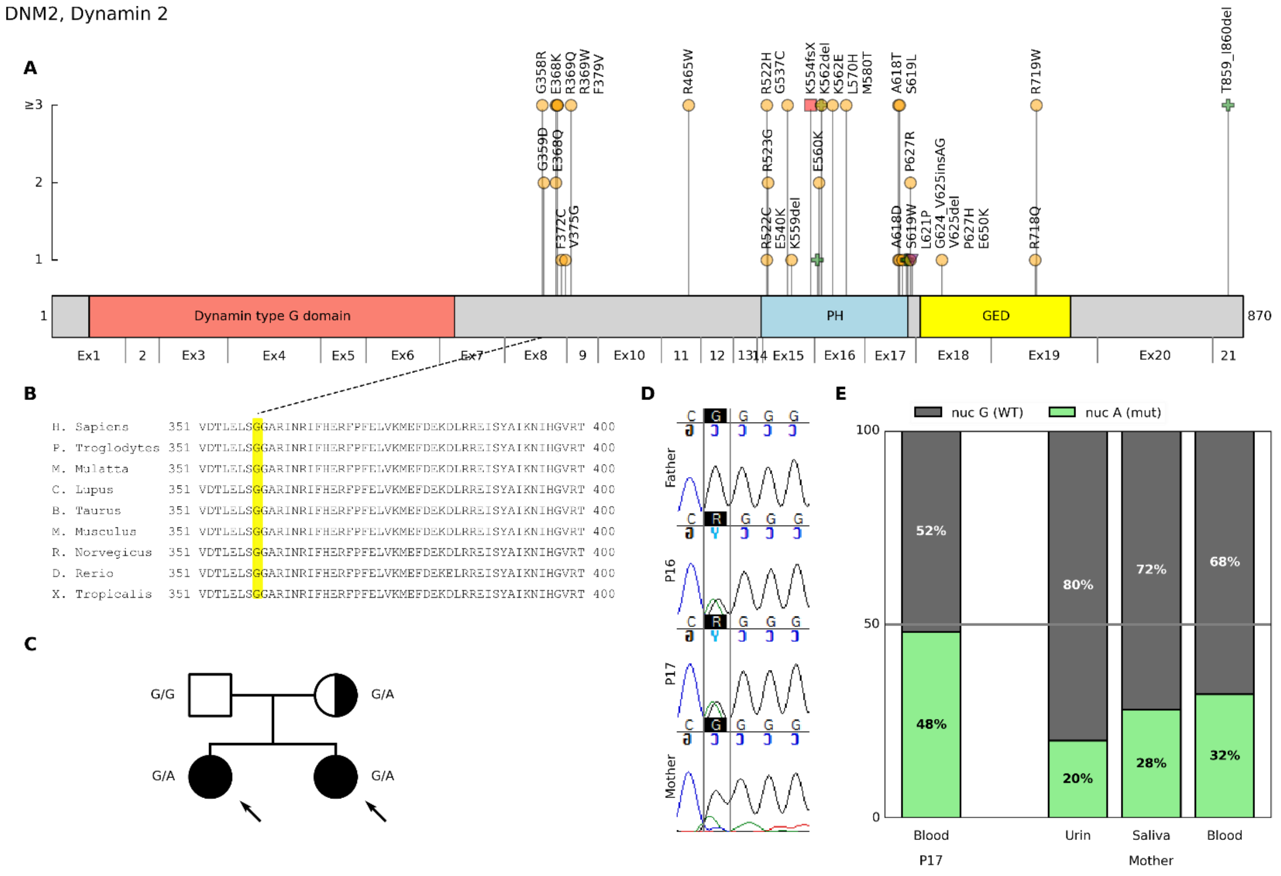

Figure 1. A) DNM2 protein with previously reported disease-causing variants. The sister-pair, P16 and P17, had the p.G358R variant. B) Conservation of the Gly358 amino acid residue (yellow shade) through species. C) Family pedigree; the parents were unaffected, while both sisters had CMT. D) Sanger sequencing was inconclusive for the mother, the father had the wild type allele, while the sisters were heterozygotes for the c.1072G>A variant. E) Minisequencing confirmed the mother to be a mosaic carrier, with a mutation load ranging from 20 to 32 % in urine, saliva, and blood.

5. Supplementary figure 2: CHD2

Chromodomain Helicase DNA-Binding Protein 2, CHD2

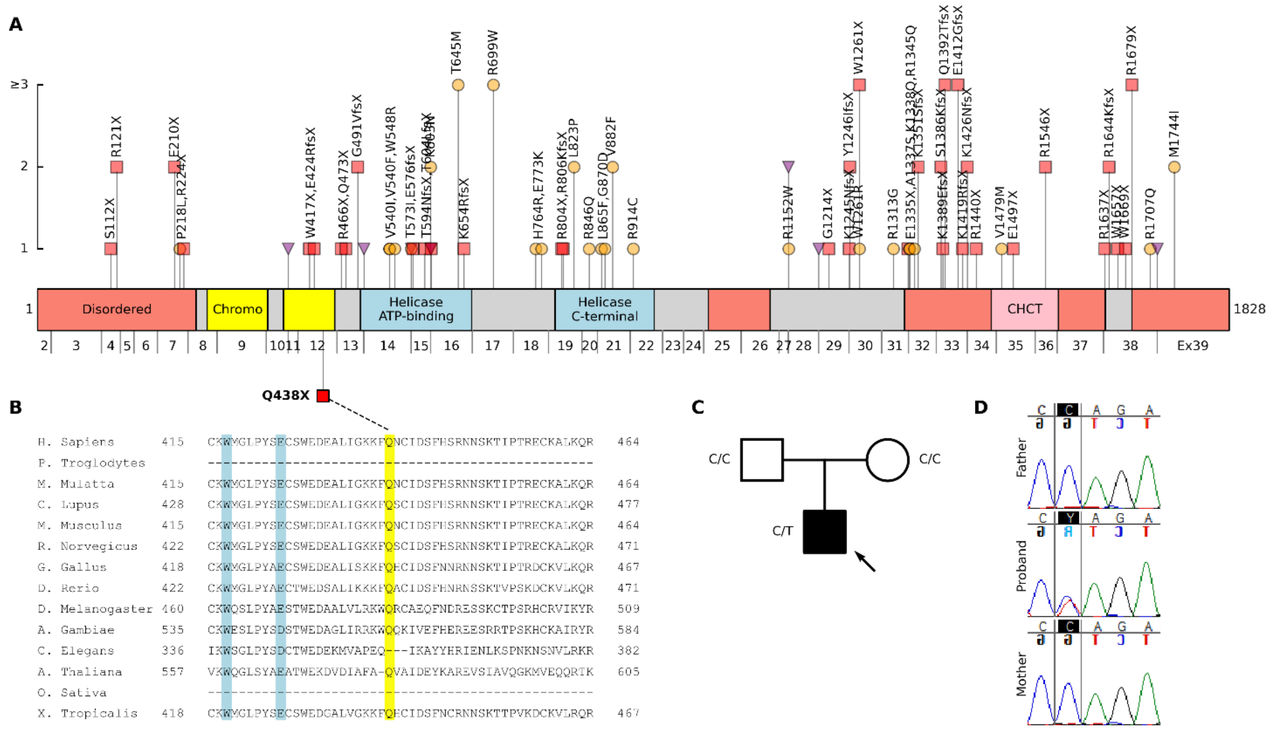

Figure 2. A) CHD2 protein with previously reported disease-causing variants, the novel p.Q438X variant is depicted below the linear graph. B) Protein conservation of the Gln438 amino acid residue (yellow shade) through species and previously reported mutations (blue shade). C) Family pedigree, the proband had a *de novo* variant. D) Sanger sequencing results confirm the inheritance pattern of c.1312C>T.

6. Supplementary figure 3: UPF3B

UPF3B Regulator of Nonsense-Mediated mRNA Decay

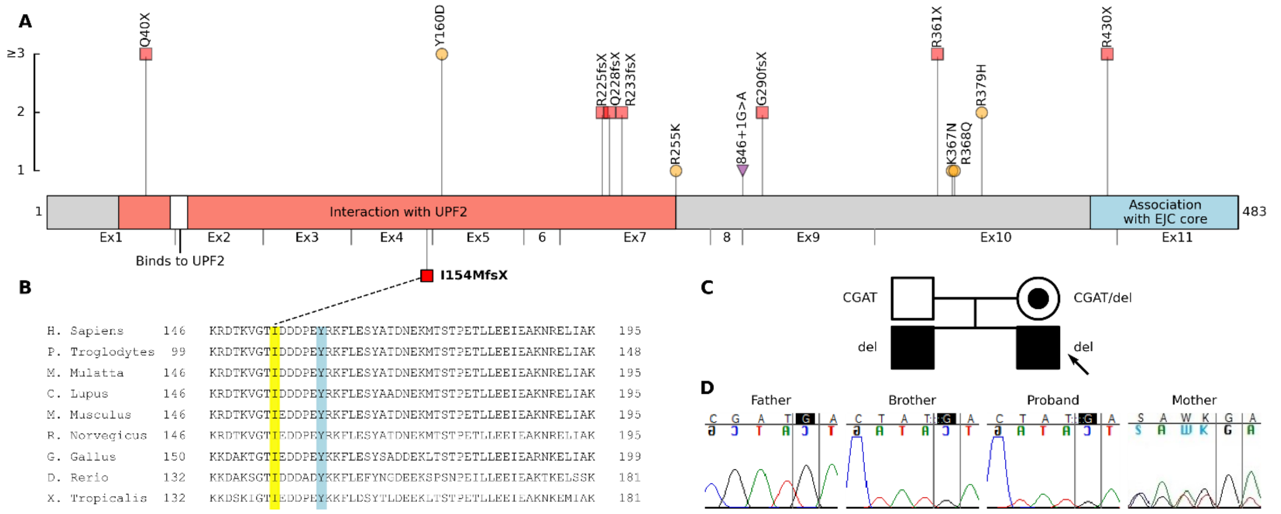

Figure 3. A) UPF3B protein with previously reported disease-causing variants, the novel p.I154MfsX variant is depicted below the linear graph. B) Protein conservation of the Ile154 amino acid residue (yellow shade) through species and previously described mutations (blue shade). C) Family pedigree, the proband and affected brother had the four-nucleotide deletion as hemizygote, while the mother was an unaffected carrier. D) Sanger sequencing results confirm the inheritance pattern of c.462_465delCGAT.

7. *Supplementary figure 4: PURA*

Purine-Rich Element-Binding Protein A, PURA

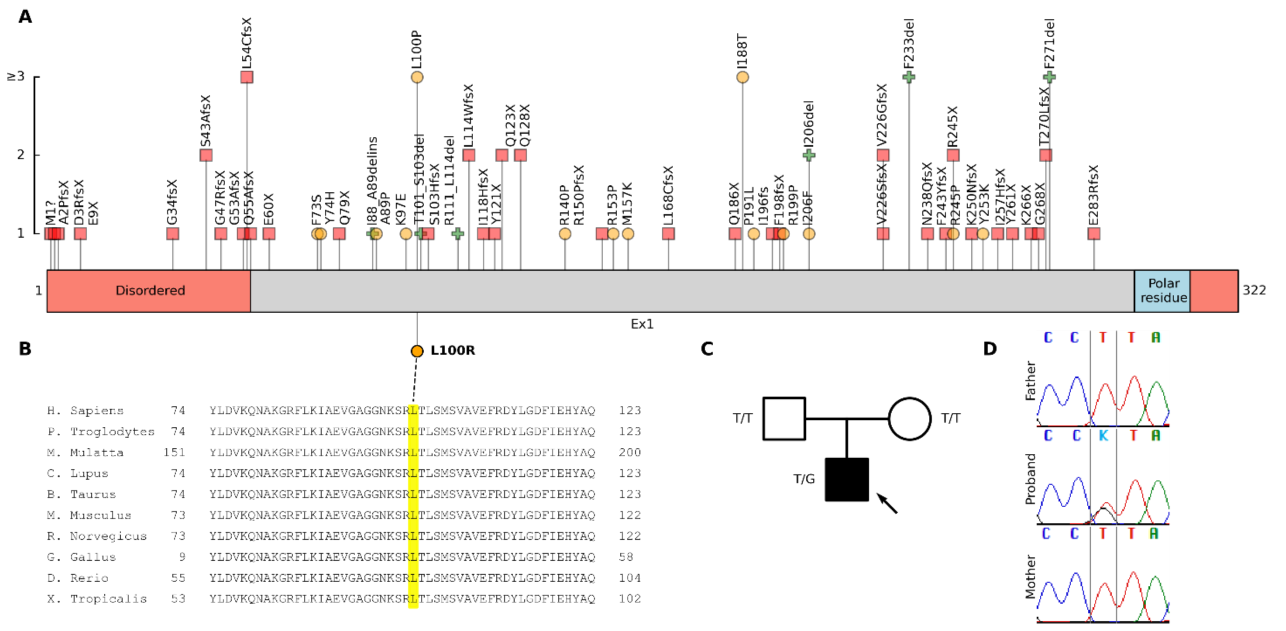

Figure 4. A) PURA protein with previously reported disease-causing variants, the novel p.L100R variant is depicted below the linear graph. B) Protein conservation of the Leu100 amino acid residue (yellow shade) through species. C) Family pedigree, the proband had a *de novo* variant. D) Sanger sequencing results confirm the inheritance pattern of c.299T>G.

8. *Supplementary figure 5: PYCR2*

Pyrroline-5-Carboxylate Reductase 2, PYCR2

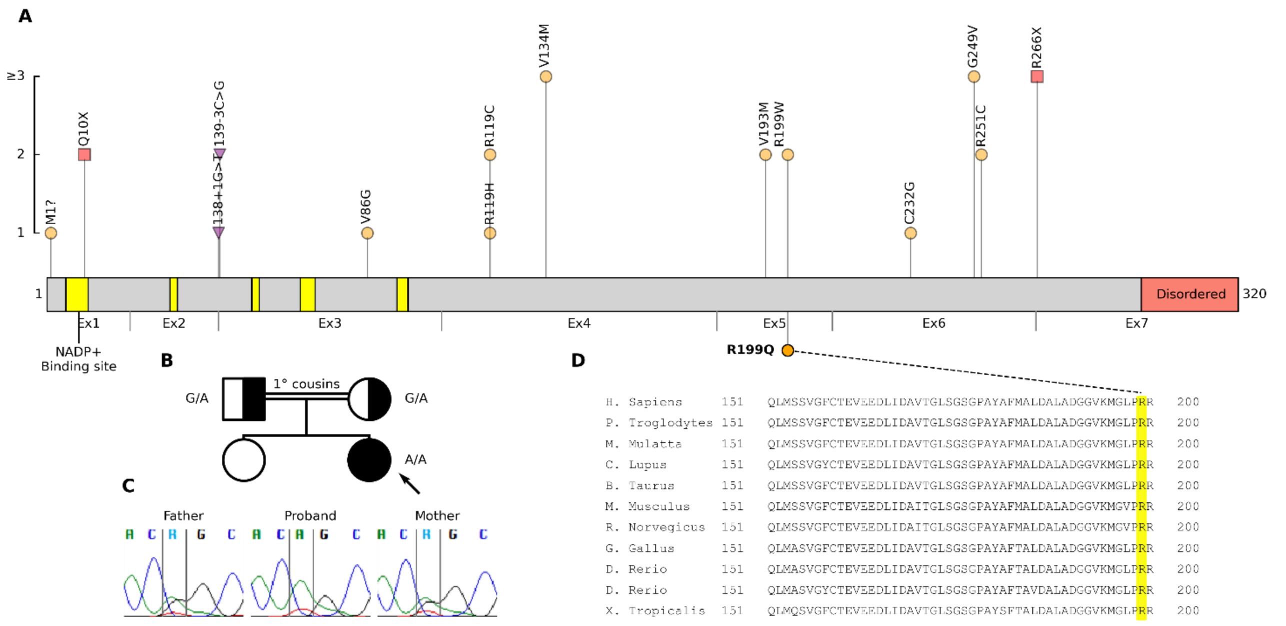

Figure 5. A) PYCR2 protein with previously reported disease-causing variants, the novel p.R199Q variant is depicted below the linear graph. B) Family pedigree, the parents were first degree cousins and heterozygote carriers of the variant, the proband homozygote. C) Sanger sequencing results confirm the inheritance pattern of c.596G>A. D) Conservation of the Arg199 amino acid residue (yellow shade) through species.

9. Gene review references

9.1 TRIT1

1. Yarham JW et al. Defective i6A37 modification of mitochondrial and cytosolic tRNAs results from pathogenic mutations in TRIT1 and its substrate tRNA. *PLoS Genet*. 2014;10(6):e1004424
2. Kernohan KD et al. Matchmaking facilitates the diagnosis of an autosomal-recessive mitochondrial disease caused by biallelic mutation of the tRNA isopentenyltransferase (TRIT1) gene. *Hum Mutat*. 2017;38(5):511-6
3. Balciuniene J et al. Use of a Dynamic Genetic Testing Approach for Childhood-Onset Epilepsy. *JAMA Netw Open*. 2019;2(4):e192129
4. Takenouchi T et al. Noninvasive diagnosis of TRIT1-related mitochondrial disorder by measuring $i^{(6)}$A37 and $ms^{(2)}i^{(6)}$A37 modifications in tRNAs from blood and urine samples. *Am J Med Genet A.* 2019;179(8):1609-14
5. Forde KM et al. Expansion of the phenotype of biallelic variants in TRIT1. *Eur J Med Genet*. 2020;63(6):103882
6. Yoo S et al. The first Korean cases of combined oxidative phosphorylation deficiency 35 with two novel TRIT1 mutations in two siblings confirmed by clinical and molecular investigation. *Brain Dev*. 2021;43(2):325

N.B. Yoo et al. 2021 report variants c.682+2T>C and p.E327K, products of a non-canonical *TRIT1* transcript (compare to p.R327X described in this study). Due to the relevance of the article, the variants are translated here according to te canonical transcript: 941+2T>C and p.E409K, respectively.

7. Yıldırım M et al. A Case of Combined Oxidative Phosphorylation Deficiency 35 Associated with a Novel Missense Variant of the TRIT1 Gene. *Mol Syndromol*. 2022;13(2):139
8. Smol T et al. TRIT1 deficiency: Two novel patients with four novel variants. *Eur J Med Genet.* 2022;65(11):104603
9. Muylle E et al. TRIT1 defect leads to a recognizable phenotype of myoclonic epilepsy, speech delay, strabismus, progressive spasticity, and normal lactate levels. *J Inherit Metab Dis*. 2022;45(6):1039

9.2 SLC1A4

1. Damseh N et al. Mutations in SLC1A4, encoding the brain serine transporter, are associated with developmental delay, microcephaly and hypomyelination. *J Med Genet*. 2015;52(8):541-547
2. Heimer G et al. SLC1A4 mutations cause a novel disorder of intellectual disability, progressive microcephaly, spasticity and thin corpus callosum. *Clin Genet*. 2015;88(4):327-335
3. Srour M et al. A homozygous mutation in SLC1A4 in siblings with severe intellectual disability and microcephaly. *Clin Genet.* 2015;88(1):e1-4
4. Conroy J et al. Novel European SLC1A4 variant: infantile spasms and population ancestry analysis. *J Hum Genet*. 2016;61(8):761-764
5. Pironti E et al. A novel SLC1A4 homozygous mutation causing congenital microcephaly, epileptic encephalopathy and spastic tetraparesis: a video-EEG and tractography - case study. *J Neurogenet*. 2018;32(4):316
6. Abdelrahman HA et al. A Novel SLC1A4 Mutation (p.Y191*) Causes Spastic Tetraplegia, Thin Corpus Callosum, and Progressive Microcephaly (SPATCCM) With Seizure Disorder. *Child Neurol Open*. 2019;6:2329048X19880647
7. Sedláčková L et al. Severe neurodevelopmental disorder with intractable seizures due to a novel SLC1A4 homozygous variant. *Eur J Med Genet.* 2021;64(9):104263
8. Sarigecili E, Bulut FD & Anlas O. A rare cause of microcephaly, thin corpus callosum and refractory epilepsy due to a novel SLC1A4 gene mutation. *Clin Neurol Neurosurg*. 2022;218:107283

9.3 ATP7A

1. Procopis P, Camakaris J & Danks DM. A mild form of Menkes steely hair syndrome. *J Pediatr*. 1981;98(1):97-99
2. Sztriha L, Janáky M, Kiss J & Buga K. Electrophysiological and 99mTc-HMPAO-SPECT studies in Menkes disease. *Brain Dev*. 1994;16(3):224-228
3. Kaler SG et al. Occipital horn syndrome and a mild Menkes phenotype associated with splice site mutations at the MNK locus. *Nat Genet.* 1994;8(2):195
4. Tümer Z et al. Identification of point mutations in 41 unrelated patients affected with Menkes disease. *Am J Hum Genet*. 1997;60(1):63
5. Murata Y et al. Mutation analysis and expression of the mottled gene in the macular mouse model of Menkes disease. *Pediatr Res*. 1997;42(4):436-442
6. Mori M & Nishimura M. A serine-to-proline mutation in the copper-transporting P-type ATPase gene of the macular mouse. *Mamm Genome*. 1997;8(6):407-410
7. Reed V & Boyd Y. Mutation analysis provides additional proof that mottled is the mouse homologue of Menkes' disease. *Hum Mol Genet.* 1997;6(3):417-423
8. Ronce N et al. A C2055T transition in exon 8 of the ATP7A gene is associated with exon skipping in an occipital horn syndrome family. *Am J Hum Genet*. 1997;61(1):233-238
9. Qi M & Byers PH. Constitutive skipping of alternatively spliced exon 10 in the ATP7A gene abolishes Golgi localization of the menkes protein and produces the occipital horn syndrome. *Hum Mol Genet*. 1998;7(3):465-469
10. Ambrosini L & Mercer JF. Defective copper-induced trafficking and localization of the Menkes protein in patients with mild and copper-treated classical Menkes disease. *Hum Mol Genet*. 1999;8(8):1547-1555
11. Tümer Z, Møller LB & Horn N. Mutation spectrum of ATP7A, the gene defective in Menkes disease. *Adv Exp Med Biol*. 1999;448:83
12. Møller LB et al. Similar splice-site mutations of the ATP7A gene lead to different phenotypes: classical Menkes disease or occipital horn syndrome. *Am J Hum Genet*. 2000;66(4):1211-1220
13. Dagenais SL, Adam AN, Innis JW & Glover TW. A novel frameshift mutation in exon 23 of ATP7A (MNK) results in occipital horn syndrome and not in Menkes disease. *Am J Hum Genet*. 2001;69(2):420-427
14. Hahn S et al. Identification of four novel mutations in classical Menkes disease and successful prenatal DNA diagnosis. *Mol Genet Metab*. 2001;73(1):86
15. Seidel J et al. Disturbed copper transport in humans. Part 1: mutations of the ATP7A gene lead to Menkes disease and occipital horn syndrome. *Cell Mol Biol (Noisy-le-grand).* 2001;47 Online Pub:OL141-8
16. Gérard-Blanluet M et al. Early development of occipital horns in a classical Menkes patient. *Am J Med Genet A*. 2004;130A(2):211-213
17. Møller LB et al. Identification and analysis of 21 novel disease-causing amino acid substitutions in the conserved part of ATP7A. *Hum Mutat*. 2005;26(2):84
18. Watanabe A & Shimizu N. Identification of three novel mutations in Japanese patients with Menkes disease and mutation screening by denaturing high performance liquid chromatography. *Pediatr Int*. 2005;47(1):1
19. Paulsen M et al. Evidence that translation reinitiation leads to a partially functional Menkes protein containing two copper-binding sites. *Am J Hum Genet.* 2006;79(2):214-229
20. Tang J et al. Functional copper transport explains neurologic sparing in occipital horn syndrome. *Genet Med.* 2006;8(11):711-718
21. Donsante A et al. Differences in ATP7A gene expression underlie intrafamilial variability in Menkes disease/occipital horn syndrome. *J Med Genet*. 2007;44(8):492-497
22. Tang J et al. Clinical outcomes in Menkes disease patients with a copper-responsive ATP7A mutation, G727R. *Mol Genet Metab*. 2008;95(3):174-181
23. Kaler SG et al. Neonatal diagnosis and treatment of Menkes disease. *N Engl J Med.* 2008;358(6):605-614
24. Møller LB & Horn N. Mutation Detection in the Menkes Gene ATP7A Using the Protein Truncation Test. *Clin Med Pathol*. 2008;1:49
25. Park HD et al. A novel ATP7A gross deletion mutation in a Korean patient with Menkes disease. *Ann Clin Lab Sci*. 2009;39(2):188-191
26. Kennerson ML et al. Missense mutations in the copper transporter gene ATP7A cause X-linked distal hereditary motor neuropathy. *Am J Hum Genet*. 2010;86(3):343-352
27. Donsante A, Johnson P, Jansen LA & Kaler SG. Somatic mosaicism in Menkes disease suggests choroid plexus-mediated copper transport to the developing brain. *Am J Med Genet A.* 2010;152A(10):2529-2534
28. Moizard MP et al. Twenty-five novel mutations including duplications in the ATP7A gene. *Clin Genet*. 2011;79(3):243-253
29. Skjørringe T, Tümer Z & Møller LB. Splice site mutations in the ATP7A gene. *PLoS One.* 2011;6(4):e18599
30. Kim YH et al. Identification of a novel mutation in the ATP7A gene in a Korean patient with Menkes disease. *J Korean Med Sci*. 2011;26(7):951-953
31. Møller LB et al. Clinical expression of Menkes disease in females with normal karyotype. *Orphanet J Rare Dis*. 2012;7:6
32. Vonk WI et al. The copper-transporting capacity of ATP7A mutants associated with Menkes disease is ameliorated by COMMD1 as a result of improved protein expression. *Cell Mol Life Sci*. 2012;69(1):149-163
33. Wang Q et al. [Clinical and ATP7A gene analysis of three infants with Menkes disease and prenatal diagnosis for a fetus at risk]. *Zhongguo Dang Dai Er Ke Za Zhi.* 2014;16(6):624-628
34. Kim D et al. Impaired osteogenesis in Menkes disease-derived induced pluripotent stem cells. *Stem Cell Res Ther*. 2015;6(1):160
35. Bansagi B et al. Phenotypic convergence of Menkes and Wilson disease. *Neurol Genet*. 2016;2(6):e119
36. Skjørringe T et al. Characterization of ATP7A missense mutants suggests a correlation between intracellular trafficking and severity of Menkes disease. *Sci Rep.* 2017;7(1):757
37. Králík L et al. Molecular Diagnostics of Copper-Transporting Protein Mutations Allows Early Onset Individual Therapy of Menkes Disease. *Folia Biol (Praha).* 2017;63(5-6):165
38. Yoganathan S et al. Menkes disease and response to copper histidine: An Indian case series. *Ann Indian Acad Neurol*. 2017;20(1):62
39. Cao B et al. Identification of novel ATP7A mutations and prenatal diagnosis in Chinese patients with Menkes disease. *Metab Brain Dis*. 2017;32(4):1123
40. Tümer Z et al. A 37-year-old Menkes disease patient-Residual ATP7A activity and early copper administration as key factors in beneficial treatment. *Clin Genet.* 2017;92(5):548

41. Bonati MT et al. A novel nonsense ATP7A pathogenic variant in a family exhibiting a variable occipital horn syndrome phenotype. *Mol Genet Metab Rep*. 2017;13:14
42. Kim MY et al. Urological Problems in Patients with Menkes Disease. *J Korean Med Sci*. 2019;34(1):e4
43. Caicedo-Herrera G et al. Novel ATP7A gene mutation in a patient with Menkes disease. *Appl Clin Genet*. 2018;11:151
44. Sharawat IK et al. Mystery Case: Tortuous hairs and tortuous blood vessels. *Neurology*. 2018;90(13):e1174-e1176
45. Woodfin T et al. Menkes disease complicated by concurrent Koolen-de Vries syndrome (17q21.31 deletion). *Mol Genet Genomic Med*. 2019;7(8):e829
46. Beyens A et al. Defining the Clinical, Molecular and Ultrastructural Characteristics in Occipital Horn Syndrome: Two New Cases and Review of the Literature. *Genes (Basel).* 2019;10(7)
47. Gualandi F et al. Report of a novel ATP7A mutation causing distal motor neuropathy. *Neuromuscul Disord*. 2019;29(10):776
48. Parad RB et al. Targeted next generation sequencing for newborn screening of Menkes disease. *Mol Genet Metab Rep*. 2020;24:100625
49. Bakkar N et al. The M1311V variant of ATP7A is associated with impaired trafficking and copper homeostasis in models of motor neuron disease. *Neurobiol Dis*. 2021;149:105228
50. Martinez-Fierro ML et al. Whole-Exome Sequencing, Proteome Landscape, and Immune Cell Migration Patterns in a Clinical Context of Menkes Disease. *Genes (Basel).* 2021;12(5)
51. Li J et al. Menkes disease diagnosed by a novel ATP7A frameshift mutation in a patient with infantile spasms-a case report. *Transl Pediatr*. 2021;10(7):1965
52. Møller LB, Mogensen M, Weaver DD & Pedersen PA. Occipital Horn Syndrome as a Result of Splice Site Mutations in ATP7A. No Activity of ATP7A Splice Variants Missing Exon 10 or Exon 15. *Front Mol Neurosci*. 2021;14:532291
53. Natera-de Benito D et al. Copper Toxicity Associated With an ATP7A-Related Complex Phenotype. Pediatr Neurol. 2021;119:40
54. Fujisawa C et al. Early clinical signs and treatment of Menkes disease. *Mol Genet Metab Rep*. 2022;31:100849
55. Guo Y, Xia W, Peng X & Shao J. Almost misdiagnosed Menkes disease: A case report. *Heliyon*. 2022;8(4):e09268
56. Panichsillaphakit E, Kwanbunbumpen T, Chomtho S & Visuthranukul C. Copper-histidine therapy in an infant with novel splice-site variant in the ATP7A gene of Menkes disease: the first experience in South East Asia and literature review. *BMJ Case Rep.* 2022;15(4)

9.4 SPAST

1. Ki CS et al. A novel missense mutation (I344K) in the SPG4gene in a Korean family with autosomal-dominant hereditary spastic paraplegia. *J Hum Genet*. 2002;47(9):473-477
2. Park SY et al. Mutation analysis of SPG4 and SPG3A genes and its implication in molecular diagnosis of Korean patients with hereditary spastic paraplegia. *Arch Neurol*. 2005;62(7):1118-1121
3. Schickel J et al. Unexpected pathogenic mechanism of a novel mutation in the coding sequence of SPG4 (spastin). *Neurology*. 2006;66(3):421-423
4. Depienne C et al. Spastin mutations are frequent in sporadic spastic paraparesis and their spectrum is different from that observed in familial cases. *J Med Genet.* 2006;43(3):259-265
5. Crippa F et al. Eight novel mutations in SPG4 in a large sample of patients with hereditary spastic paraplegia. *Arch Neurol*. 2006;63(5):750-755
6. Bertelli M et al. Identification of a novel mutation in the spastin gene (SPG4) in an Italian family with hereditary spastic paresis. *Panminerva Med*. 2006;48(3):193-197
7. Magariello A et al. Novel spastin (SPG4) mutations in Italian patients with hereditary spastic paraplegia. *Neuromuscul Disord*. 2006;16(6):387-390
8. Fukunaga M et al. [Late-onset sporadic case of SPG4 (1726T>C mutant) accompanied by polyneuropathy with diabetes mellitus]. *Rinsho Shinkeigaku*. 2007;47(6):359-361
9. Schickel J et al. Isoform-specific increase of spastin stability by N-terminal missense variants including intragenic modifiers of SPG4 hereditary spastic paraplegia. *Eur J Neurol*. 2007;14(12):1322-1328
10. Depienne C et al. Exon deletions of SPG4 are a frequent cause of hereditary spastic paraplegia. *J Med Genet*. 2007;44(4):281-284
11. Depienne C et al. A de novo SPAST mutation leading to somatic mosaicism is associated with a later age at onset in HSP. *Neurogenetics*. 2007;8(3):231-233
12. Blair MA et al. Infantile onset of hereditary spastic paraplegia poorly predicts the genotype. *Pediatr Neurol*. 2007;36(6):382-386
13. Ribaï P et al. Mental deficiency in three families with SPG4 spastic paraplegia. *Eur J Hum Genet*. 2008;16(1):97
14. Orlacchio A et al. Silver syndrome variant of hereditary spastic paraplegia: A locus to 4p and allelism with SPG4. *Neurology*. 2008;70(21):1959-1966
15. Pantakani DV et al. Compound heterozygosity in the SPG4 gene causes hereditary spastic paraplegia. *Clin Genet*. 2008;73(3):268-272
16. Shoukier M et al. Expansion of mutation spectrum, determination of mutation cluster regions and predictive structural classification of SPAST mutations in hereditary spastic paraplegia. *Eur J Hum Genet*. 2009;17(2):187-194
17. Svenstrup K et al. Sequence variants in SPAST, SPG3A and HSPD1 in hereditary spastic paraplegia. *J Neurol Sci*. 2009;284(1-2):90-95
18. Lau KK, Ching CK, Mak CM & Chan YW. Hereditary spastic paraplegias. *Hong Kong Med J*. 2009;15(3):217-220
19. Alvarez V et al. Mutational spectrum of the SPG4 (SPAST) and SPG3A (ATL1) genes in Spanish patients with hereditary spastic paraplegia. *BMC Neurol*. 2010;10:89
20. Magariello A et al. Mutation analysis of the SPG4 gene in Italian patients with pure and complicated forms of spastic paraplegia. *J Neurol Sci*. 2010;288(1-2):96
21. Braschinsky M et al. Unique spectrum of SPAST variants in Estonian HSP patients: presence of benign missense changes but lack of exonic rearrangements. *BMC Neurol*. 2010;10:17
22. Rudenskaia GE et al. [Hereditary spastic paraplegia type 4 (SPG4): clinical and molecular-genetic characteristics]. *Zh Nevrol Psikhiatr Im S S Korsakova*. 2010;110(6):12-19
23. Lim JS et al. A novel splicing mutation (c.870+3A>G) in SPG4 in a Korean family with hereditary spastic paraplegia. *J Neurol Sci*. 2010;290(1-2):186-189
24. Fei QZ et al. Two novel mutations in the Spastin gene of Chinese patients with hereditary spastic paraplegia. *Eur J Neurol*. 2011;18(9):1194-1196
25. McCorquodale DS et al. Mutation screening of spastin, atlastin, and REEP1 in hereditary spastic paraplegia. *Clin Genet*. 2011;79(6):523-530
26. Klimpe S et al. Evaluating the effect of spastin splice mutations by quantitative allele-specific expression assay. *Eur J Neurol*. 2011;18(1):99
27. Battini R et al. Clinical and genetic findings in a series of Italian children with pure hereditary spastic paraplegia. *Eur J Neurol*. 2011;18(1):150-157
28. Vandebona H, Kerr NP, Liang C & Sue CM. SPAST mutations in Australian patients with hereditary spastic paraplegia. *Intern Med J*. 2012;42(12):1342-1347
29. Loureiro JL et al. Autosomal dominant spastic paraplegias: a review of 89 families resulting from a portuguese survey. *JAMA Neurol*. 2013;70(4):481-487
30. Guthrie G et al. The neurological and ophthalmological manifestations of SPG4-related hereditary spastic paraplegia. *J Neurol*. 2013;260(3):906-909
31. Yang JW et al. Hereditary Spastic Paraplegia with a Novel SPAST Mutation Misdiagnosed with Subacute Combined Degeneration. *Exp Neurobiol*. 2013;22(2):128-131
32. Lan MY et al. High frequency of SPG4 in Taiwanese families with autosomal dominant hereditary spastic paraplegia. *BMC Neurol*. 2014;14:216
33. Kim TH et al. Mutation analysis of SPAST, ATL1, and REEP1 in Korean Patients with Hereditary Spastic Paraplegia. *J Clin Neurol*. 2014;10(3):257-261
34. Wei QQ et al. Spastin mutation screening in Chinese patients with pure hereditary spastic paraplegia. *Parkinsonism Relat Disord*. 2014;20(8):845-849
35. Aulitzky A et al. A complex form of hereditary spastic paraplegia in three siblings due to somatic mosaicism for a novel SPAST mutation in the mother. *J Neurol Sci*. 2014;347(1-2):352-355
36. Havlicek S et al. Gene dosage-dependent rescue of HSP neurite defects in SPG4 patients' neurons. *Hum Mol Genet*. 2014;23(10):2527-2541
37. Rezende TJ et al. Multimodal MRI-based study in patients with SPG4 mutations. *PLoS One*. 2015;10(2):e0117666
38. Zhao N et al. Mutation analysis of four Chinese families with pure hereditary spastic paraplegia: pseudo- X-linked dominant inheritance and male lethality due to a novel ATL1 mutation. *Genet Mol Res*. 2015;14(4):14690-14697
39. Polymeris AA et al. A series of Greek children with pure hereditary spastic paraplegia: clinical features and genetic findings. *J Neurol*. 2016;263(8):1604-1611
40. de Souza PV et al. Infantile-onset ascending spastic paraplegia phenotype associated with SPAST mutation. *J Neurol Sci*. 2016;371:34
41. Lynch DS et al. Hereditary spastic paraplegia in Greece: characterisation of a previously unexplored population using next-generation sequencing. *Eur J Hum Genet*. 2016;24(6):857-863
42. Hauser S et al. Establishment of SPAST mutant induced pluripotent stem cells (iPSCs) from a hereditary spastic paraplegia (HSP) patient. *Stem Cell Res*. 2016;17(3):485
43. Chelban V et al. Truncating mutations in SPAST patients are associated with a high rate of psychiatric comorbidities in hereditary spastic paraplegia. *J Neurol Neurosurg Psychiatry*. 2017;88(8):681
44. Burguez D et al. Clinical and molecular characterization of hereditary spastic paraplegias: A next-generation sequencing panel approach. *J Neurol Sci*. 2017;383:18
45. Kawarai T et al. Spastic paraplegia type 4: A novel SPAST splice site donor mutation and expansion of the phenotype variability. *J Neurol Sci*. 2017;380:92
46. Morais S et al. Massive sequencing of 70 genes reveals a myriad of missing genes or mechanisms to be uncovered in hereditary spastic paraplegias. *Eur J Hum Genet*. 2017;25(11):1217
47. Gillespie MK, Humphreys P, McMillan HJ & Boycott KM. Association of Early-Onset Spasticity and Risk for Cognitive Impairment With Mutations at Amino Acid 499 in SPAST. *J Child Neurol*. 2018;33(5):329
48. Parodi L et al. Spastic paraplegia due to SPAST mutations is modified by the underlying mutation and sex. *Brain*. 2018;141(12):3331
49. Takezawa Y et al. Genomic analysis identifies masqueraders of full-term cerebral palsy. *Ann Clin Transl Neurol*. 2018;5(5):538
50. Duz MB et al. Three novel mutations in 20 patients with hereditary spastic paraparesis. *Neurol Sci*. 2018;39(9):1551
51. Schieving JH et al. De novo SPAST mutations may cause a complex SPG4 phenotype. *Brain*. 2019;142(7):e31
52. Zhu Z et al. Novel mutations in the SPAST gene cause hereditary spastic paraplegia. *Parkinsonism Relat Disord*. 2019;69:125

53. Kadnikova VA et al. Mutational Spectrum of Spast (Spg4) and Atl1 (Spg3a) Genes In Russian Patients With Hereditary Spastic Paraplegia. *Sci Rep*. 2019;9(1):14412
54. Zhao M et al. Genetic and Clinical Profile of Chinese Patients with Autosomal Dominant Spastic Paraplegia. *Mol Diagn Ther*. 2019;23(6):781
55. Yang J, Seo JY, Lee KW & Park HM. Novel Pathogenic Variant of SPAST (c.1413+4A>G) in a Patient with Hereditary Spastic Paraplegia. *J Clin Neurol*. 2019;15(1):120
56. Elert-Dobkowska E et al. Next-generation sequencing study reveals the broader variant spectrum of hereditary spastic paraplegia and related phenotypes. *Neurogenetics*. 2019;20(1):27
57. Klimkowicz-Mrowiec A et al. Case report on novel mutation in SPAST gene in Polish family with spastic paraplegia. *BMC Neurol*. 2019;19(1):322
58. Ogasawara M et al. A p.Arg499His Mutation in SPAST Is Associated with Infantile Onset Ascending Spastic Paralysis Complicated with Dysarthria and Anarthria. *Neuropediatrics*. 2019;50(6):391
59. Almomen M et al. High diagnostic yield and novel variants in very late-onset spasticity. *J Neurogenet*. 2019;33(1):27
60. Rucco R et al. Mutations in the SPAST gene causing hereditary spastic paraplegia are related to global topological alterations in brain functional networks. *Neurol Sci*. 2019;40(5):979
61. Rehbach K et al. Multiparametric rapid screening of neuronal process pathology for drug target identification in HSP patient-specific neurons. *Sci Rep*. 2019;9(1):9615
62. Oberoi K, Grewal KS & Reddy Peddareddygari L. Complicated SPG4 presenting with recurrent urinary tract infection. *J Community Hosp Intern Med Perspect*. 2020;10(4):369
63. Bertran Recasens B, Figueras Aguirre G, Aznar-Lain G & Rubio MA. A novel pathogenic variant of the SPAST gene in a Spanish family with hereditary spastic paraplegia. *Neurologia* (Engl Ed). 2020;35(5):340
64. Nan H et al. A Japanese SPG4 Patient with a Confirmed De Novo Mutation of the SPAST Gene. *Intern Med*. 2020;59(18):2311
65. Lin JZ et al. Cortical Damage Associated With Cognitive and Motor Impairment in Hereditary Spastic Paraplegia: Evidence of a Novel SPAST Mutation. *Front Neurol*. 2020;11:399
66. Angelini C et al. Evidence of mosaicism in SPAST variant carriers in four French families. *Eur J Hum Genet*. 2021;29(7):1158
67. Giordani GM et al. Clinical and molecular characterization of a large cohort of childhood onset hereditary spastic paraplegias. *Sci Rep*. 2021;11(1):22248
68. Nan H, Shiraku H, Mizuno T & Takiyama Y. A p.Arg499His mutation in SPAST is associated with infantile-onset complicated spastic paraplegia: a case report and review of the literature. *BMC Neurol*. 2021;21(1):439
69. Abe-Hatano C et al. Whole genome sequencing of 45 Japanese patients with intellectual disability. *Am J Med Genet A*. 2021;185(5):1468
70. Kwong AK et al. Exome sequencing in paediatric patients with movement disorders. *Orphanet J Rare Dis*. 2021;16(1):32
71. Wang C et al. The investigation of genetic and clinical features in patients with hereditary spastic paraplegia in central-Southern China. *Mol Genet Genomic Med*. 2021;9(5):e1627
72. Suchowersky O et al. Hereditary spastic paraplegia initially diagnosed as cerebral palsy. *Clin Park Relat Disord*. 2021;5:100114
73. Nagai T et al. Coexistence of Hereditary Spastic Paraplegia Type 4 and Narcolepsy: A Case Report. *Case Rep Neurol*. 2021;13(1):84
74. Morikawa T et al. A Japanese hereditary spastic paraplegia family with a rare nonsynonymous variant in the SPAST gene. *Hum Genome Var*. 2021;8(1):21
75. Wang J et al. Clinical and genetic spectrum of hereditary spastic paraplegia in Chinese children. *Dev Med Child Neurol*. 2022
76. Rossi S et al. Clinical-Genetic Features Influencing Disability in Spastic Paraplegia Type 4: A Cross-sectional Study by the Italian DAISY Network. *Neurol Genet*. 2022;8(2):e664
77. Varghaei P et al. Genetic, structural and clinical analysis of spastic paraplegia 4. *Parkinsonism Relat Disord*. 2022;98:62
78. Mo A et al. Early-Onset and Severe Complex Hereditary Spastic Paraplegia Caused by De Novo Variants in SPAST. *Mov Disord.* 2022;37(12):2440
79. Rizo T et al. Store-operated calcium entry is reduced in spastin-linked hereditary spastic paraplegia. *Brain*. 2022;145(9):3131
80. Perić S et al. Phenotypic and Genetic Heterogeneity of Adult Patients with Hereditary Spastic Paraplegia from Serbia. *Cells*. 2022;11(18)
81. ElSheikh RH, Aravindhan A, Boysen S & Veerapandiyan A. Infantile-Onset Complex Hereditary Spastic Paraplegia Due to a Novel Mutation in SPAST Gene. *Pediatr Neurol*. 2022;134:71
82. Chen R et al. A Novel SPAST Mutation Results in Spastin Accumulation and Defects in Microtubule Dynamics. *Mov Disord*. 2022;37(3):598
83. Chopra M et al. Mendelian etiologies identified with whole exome sequencing in cerebral palsy. *Ann Clin Transl Neurol*. 2022;9(2):193
84. Panwala TF et al. Childhood-Onset Hereditary Spastic Paraplegia (HSP): A Case Series and Review of Literature. *Pediatr Neurol*. 2022;130:7
85. Shi Y et al. Clinical Features and Genetic Spectrum of Patients With Clinically Suspected Hereditary Progressive Spastic Paraplegia. *Front Neurol*. 2022;13:872927
86. Nan H et al. A p.Glu420Gln mutation in SPAST is associated with infantile onset spastic paraplegia complicated by cerebella ataxia, epilepsy, peripheral neuropathy, and hypoplasia of the corpus callosum. *Neurol Sci*. 2022;43(3):2123

9.5 CHD2

1. Rauch A et al. Range of genetic mutations associated with severe non-syndromic sporadic intellectual disability: an exome sequencing study. *Lancet*. 2012;380(9854):1674-1682
2. Suls A et al. De novo loss-of-function mutations in CHD2 cause a fever-sensitive myoclonic epileptic encephalopathy sharing features with Dravet syndrome. *Am J Hum Genet*. 2013;93(5):967-975
3. Carvill GL et al. Targeted resequencing in epileptic encephalopathies identifies de novo mutations in CHD2 and SYNGAP1. *Nat Genet*. 2013;45(7):825-830
4. Epi4K Consortium et al. De novo mutations in epileptic encephalopathies. *Nature*. 2013;501(7466):217-221
5. Hamdan FF et al. De novo mutations in moderate or severe intellectual disability. *PLoS Genet*. 2014;10(10):e1004772
6. Galizia EC et al. CHD2 variants are a risk factor for photosensitivity in epilepsy. *Brain*. 2015;138(Pt 5):1198-1207
7. Thomas RH et al. CHD2 myoclonic encephalopathy is frequently associated with self-induced seizures. *Neurology*. 2015;84(9):951-958
8. Trivisano M et al. CHD2 mutations are a rare cause of generalized epilepsy with myoclonic-atonic seizures. *Epilepsy Behav*. 2015;51:53-56
9. Lebrun N et al. Autism spectrum disorder recurrence, resulting of germline mosaicism for a CHD2 gene missense variant. *Clin Genet*. 2017;92(6):669
10. Wang Y et al. Genetic Variants Identified from Epilepsy of Unknown Etiology in Chinese Children by Targeted Exome Sequencing. *Sci Rep*. 2017;7:40319
11. Ortega-Moreno L et al. Molecular diagnosis of patients with epilepsy and developmental delay using a customized panel of epilepsy genes. *PLoS One*. 2017;12(11):e0188978
12. Rim JH et al. Efficient strategy for the molecular diagnosis of intractable early-onset epilepsy using targeted gene sequencing. *BMC Med Genomics*. 2018;11(1):6
13. Staněk D et al. Detection rate of causal variants in severe childhood epilepsy is highest in patients with seizure onset within the first four weeks of life. *Orphanet J Rare Dis*. 2018;13(1):71
14. Petersen AK, Streff H, Tokita M & Bostwick BL. The first reported case of an inherited pathogenic CHD2 variant in a clinically affected mother and daughter. *Am J Med Genet A.* 2018;176(7):1667
15. Tsang MH et al. Exome sequencing identifies molecular diagnosis in children with drug-resistant epilepsy. *Epilepsia Open*. 2019;4(1):63
16. Liu J et al. Novel and de novo mutations in pediatric refractory epilepsy. *Mol Brain.* 2018;11(1):48
17. Turner TN et al. Sex-Based Analysis of De Novo Variants in Neurodevelopmental Disorders. *Am J Hum Genet*. 2019;105(6):1274
18. Cabrera-Salcedo C et al. Targeted Searches of the Electronic Health Record and Genomics Identify an Etiology in Three Patients with Short Stature and High IGF-I Levels. *Horm Res Paediatr*. 2019;92(3):186
19. Chen J et al. CHD2-related epilepsy: novel mutations and new phenotypes. *Dev Med Child Neurol*. 2020;62(5):647
20. Poisson A et al. Chromatin remodeling dysfunction extends the etiological spectrum of schizophrenia: a case report. *BMC Med Genet*. 2020;21(1):10
21. Wang T et al. Large-scale targeted sequencing identifies risk genes for neurodevelopmental disorders. *Nat Commun*. 2020;11(1):4932
22. Menon RN et al. Drug-resistant 'Non-Lesional' Visual Sensitive Epilepsies of Childhood - Electroclinical Phenotype-Genotype Associations. *Neurol India*. 2021;69(6):1701
23. Feng W et al. Clinical analysis of CHD2 gene mutations in pediatric patients with epilepsy. *Pediatr Investig*. 2022;6(2):93
24. Luo X et al. Clinical Study of 8 Cases of CHD2 Gene Mutation-Related Neurological Diseases and Their Mechanisms. *Front Cell Dev Biol*. 2022;10:853127
25. Wang X et al. Novel Loss-of-Function Variants in CHD2 Cause Childhood-Onset Epileptic Encephalopathy in Chinese Patients. *Genes (Basel)*. 2022;13(5)
26. Zhu L et al. A Novel Variant of the CHD2 Gene Associated With Developmental Delay and Myoclonic Epilepsy. *Front Genet*. 2022;13:761178
27. Niu Y et al. Genetic and phenotypic spectrum of Chinese patients with epilepsy and photosensitivity. *Front Neurol*. 2022;13:907228

9.6 UPF3B

1. Tarpey PS et al. Mutations in UPF3B, a member of the nonsense-mediated mRNA decay complex, cause syndromic and nonsyndromic mental retardation. *Nat Genet*. 2007;39(9):1127-1133
2. Addington AM et al. A novel frameshift mutation in UPF3B identified in brothers affected with childhood onset schizophrenia and autism spectrum disorders. *Mol Psychiatry*. 2011;16(3):238-239
3. Laumonnier F et al. Mutations of the UPF3B gene, which encodes a protein widely expressed in neurons, are associated with nonspecific mental retardation with or without autism. *Mol Psychiatry*. 2010;15(7):767-776
4. Lynch SA et al. Broadening the phenotype associated with mutations in UPF3B: two further cases with renal dysplasia and variable developmental delay. *Eur J Med Genet*. 2012;55(8-9):476-479
5. Szyszka P et al. A nonconservative amino acid change in the UPF3B gene in a patient with schizophrenia. *Psychiatr Genet*. 2012;22(3):150-151

6. Xu X et al. Exome sequencing identifies UPF3B as the causative gene for a Chinese non-syndrome mental retardation pedigree. *Clin Genet*. 2013;83(6):560-564
7. Tzschach A et al. Next-generation sequencing in X-linked intellectual disability. *Eur J Hum Genet*. 2015;23(11):1513-1518
8. Chérot E et al. Using medical exome sequencing to identify the causes of neurodevelopmental disorders: Experience of 2 clinical units and 216 patients. *Clin Genet*. 2018;93(3):567
9. Tejada MI et al. Molecular and Clinical Characterization of a Novel Nonsense Variant in Exon 1 of the UPF3B Gene Found in a Large Spanish Basque Family (MRX82). *Front Genet.* 2019;10:1074

9.7 PURA

1. Hunt D et al. Whole exome sequencing in family trios reveals de novo mutations in PURA as a cause of severe neurodevelopmental delay and learning disability. *J Med Genet*. 2014;51(12):806-813
2. Lalani SR et al. Mutations in PURA cause profound neonatal hypotonia, seizures, and encephalopathy in 5q31.3 microdeletion syndrome. *Am J Hum Genet*. 2014;95(5):579-583
3. Tanaka AJ et al. De novo mutations in PURA are associated with hypotonia and developmental delay. *Cold Spring Harb Mol Case Stud*. 2015;1(1):a000356
4. Rezkalla J, Von Wald T & Hansen KA. Premature Thelarche and the PURA Syndrome. *Obstet Gynecol*. 2017;129(6):1037
5. Eldomery MK et al. Lessons learned from additional research analyses of unsolved clinical exome cases. *Genome Med*. 2017;9(1):26
6. Okamoto N, Nakao H, Niihori T & Aoki Y. Patient with a novel purine-rich element binding protein A mutation. *Congenit Anom (Kyoto).* 2017;57(6):201
7. Reijnders MRF et al. PURA syndrome: clinical delineation and genotype-phenotype study in 32 individuals with review of published literature. *J Med Genet.* 2018;55(2):104
8. Lee BH et al. Expanding the neurodevelopmental phenotype of PURA syndrome. *Am J Med Genet A*. 2018;176(1):56
9. Mayorga L, Gamboni B, Mampel A & Roqué M. A frame-shift deletion in the PURA gene associates with a new clinical finding: Hypoglycorrhachia. Is GLUT1 a new PURA target? *Mol Genet Metab*. 2018;123(3):331
10. Qiao Y et al. Exome sequencing identified a de novo mutation of PURA gene in a patient with familial Xp22.31 microduplication. *Eur J Med Genet*. 2019;62(2):103
11. Reuter MS et al. The Cardiac Genome Clinic: implementing genome sequencing in pediatric heart disease. *Genet Med*. 2020;22(6):1015
12. Rodríguez-García ME et al. A novel de novo mutation in the PURA gene associated with a new clinical finding: large brainstem. *J Genet*. 2020;99
13. Boczek NJ et al. Expansion of PURA-Related Phenotypes and Discovery of a Novel PURA Variant: A Case Report. *Child Neurol Open*. 2020;7:2329048X20955003
14. Jezela-Stanek A et al. The phenotype-driven computational analysis yields clinical diagnosis for patients with atypical manifestations of known intellectual disability syndromes. *Mol Genet Genomic Med*. 2020;8(9):e1263
15. Trau SP & Pizoli CE. PURA Syndrome and Myotonia. *Pediatr Neurol*. 2020;104:62
16. Lin SJ et al. Complex Movement Disorders in a Boy with PURA Syndrome. *Mov Disord Clin Pract*. 2021;8(7):1137
17. Liu Y et al. Neonatal PURA syndrome: a case report and literature review. *Transl Pediatr*. 2021;10(1):194
18. Cinquina V et al. Expanding the PURA syndrome phenotype: A child with the recurrent PURA p.(Phe233del) pathogenic variant showing similarities with cutis laxa. *Mol Genet Genomic Med*. 2021;9(1):e1562
19. Kwong AK et al. Exome sequencing in paediatric patients with movement disorders. *Orphanet J Rare Dis*. 2021;16(1):32
20. Spangenberg L et al. Novel frameshift mutation in PURA gene causes severe encephalopathy of unclear cause. *Mol Genet Genomic Med*. 2021;9(5):e1622
21. Mishra S, Girisha KM & Shukla A. Expanding the phenotype of PURA-related neurodevelopmental disorder: a close differential diagnosis of infantile hypotonia with psychomotor retardation and characteristic facies. *Clin Dysmorphol.* 2021;30(1):1
22. Nogueira M et al. PURA syndrome in a child with severe developmental delay: a challenging diagnosis. *Rev Neurol*. 2022;74(5):170
23. Fukuda Y et al. Expanding the PURA syndrome phenotype with manifestations in a Japanese female patient. *Hum Genome Var*. 2022;9(1):11
24. Wyrebek R et al. Hypotonic infant with PURA syndrome-related channelopathy successfully treated with pyridostigmine. *Neuromuscul Disord*. 2022;32(2):166

9.8 PYCR2

1. Nakayama T et al. Mutations in PYCR2, Encoding Pyrroline-5-Carboxylate Reductase 2, Cause Microcephaly and Hypomyelination. *Am J Hum Genet*. 2015;96(5):709-719
2. Zaki MS et al. PYCR2 Mutations cause a lethal syndrome of microcephaly and failure to thrive. *Ann Neurol*. 2016;80(1):59
3. Meng L et al. Homozygous variants in pyrroline-5-carboxylate reductase 2 (PYCR2) in patients with progressive microcephaly and hypomyelinating leukodystrophy. *Am J Med Genet A*. 2017;173(2):460
4. Bick D et al. Successful Application of Whole Genome Sequencing in a Medical Genetics Clinic. *J Pediatr Genet*. 2017;6(2):61
5. Escande-Beillard N et al. Loss of PYCR2 Causes Neurodegeneration by Increasing Cerebral Glycine Levels via SHMT2. *Neuron*. 2020;107(1):82-94.e6
6. Afroze B & Mercimek-Andrews S. Pyrroline-5-Carboxylate Reductase 2 Deficiency: A New Case and Review of the Literature. *Can J Neurol Sci*. 2020;47(2):280
7. Manaspon C et al. Expanding the genotypic spectrum of PYCR2 and a common ancestry in Thai patients with hypomyelinating leukodystrophy 10. *Am J Med Genet A*. 2021;185(10):3068
8. Srivastava P, Mishra AK & Sarkar N. PYCR2 Mutation Causing Hypomyelination and Microcephaly in an Indian Child. *Cureus*. 2021;13(4):e14661

9.9 DNM2

1. Gallardo E et al. Magnetic resonance imaging findings of leg musculature in Charcot-Marie-Tooth disease type 2 due to dynamin 2 mutation. *J Neurol*. 2008;255(7):986-992
2. Bitoun M et al. A novel mutation in the dynamin 2 gene in a Charcot-Marie-Tooth type 2 patient: clinical and pathological findings. *Neuromuscul Disord.* 2008;18(4):334-338
3. Bitoun M et al. Dynamin 2 mutations associated with human diseases impair clathrin-mediated receptor endocytosis. *Hum Mutat.* 2009;30(10):1419-1427
4. Bevilacqua JA et al. "Necklace" fibers, a new histological marker of late-onset MTM1-related centronuclear myopathy. *Acta Neuropathol*. 2009;117(3):283-291
5. Jungbluth H et al. Centronuclear myopathy with cataracts due to a novel dynamin 2 (DNM2) mutation. *Neuromuscul Disord*. 2010;20(1):49
6. Melberg A et al. Adult course in dynamin 2 dominant centronuclear myopathy with neonatal onset. *Neuromuscul Disord*. 2010;20(1):53-56
7. Liewluck T, Lovell TL, Bite AV & Engel AG. Sporadic centronuclear myopathy with muscle pseudohypertrophy, neutropenia, and necklace fibers due to a DNM2 mutation. *Neuromuscul Disord*. 2010;20(12):801-804
8. Lin KP et al. The mutational spectrum in a cohort of Charcot-Marie-Tooth disease type 2 among the Han Chinese in Taiwan. *PLoS One*. 2011;6(12):e29393
9. Hanisch F et al. Phenotype variability and histopathological findings in centronuclear myopathy due to DNM2 mutations. *J Neurol*. 2011;258(6):1085-1090
10. Haberlová J et al. Phenotypic variability in a large Czech family with a dynamin 2-associated Charcot-Marie-Tooth neuropathy. *J Neurogenet*. 2011;25(4):182-188
11. Catteruccia M et al. Centronuclear myopathy related to dynamin 2 mutations: clinical, morphological, muscle imaging and genetic features of an Italian cohort. *Neuromuscul Disord*. 2013;23(3):229-238
12. Koutsopoulos OS et al. Dynamin 2 homozygous mutation in humans with a lethal congenital syndrome. *Eur J Hum Genet*. 2013;21(6):637-642
13. Chen T et al. Clinical, pathological, and genetic features of dynamin-2-related centronuclear myopathy in China. *Neurol Sci*. 2015;36(5):735-741
14. Sambuughin N et al. Adult-onset autosomal dominant spastic paraplegia linked to a GTPase-effector domain mutation of dynamin 2. *BMC Neurol.* 2015;15:223
15. Abath Neto O et al. DNM2 mutations in a cohort of sporadic patients with centronuclear myopathy. *Genet Mol Biol*. 2015;38(2):147-151
16. Auer F et al. Activation-induced cytidine deaminase prevents pro-B cell acute lymphoblastic leukemia by functioning as a negative regulator in Rag1 deficient pro-B cells. *Oncotarget.* 2017;8(44):75797
17. Chen S et al. Phenotype variability and histopathological findings in patients with a novel DNM2 mutation. *Neuropathology.* 2018;38(1):34
18. Dudhal S et al. Development of versatile allele-specific siRNAs able to silence all the dominant dynamin 2 mutations. *Mol Ther Nucleic Acids*. 2022;29:733
